# The DNA-dependent protein kinase catalytic subunit exacerbates endotoxemia-induced myocardial microvascular injury by disrupting the MOTS-c/JNK pathway and inducing profilin-mediated lamellipodia degradation

**DOI:** 10.7150/thno.92650

**Published:** 2024-02-04

**Authors:** Rongjun Zou, Wanting Shi, Xing Chang, Miao Zhang, Songtao Tan, Ruibing Li, Hao Zhou, Yukun Li, Ge Wang, Weihui Lv, Xiaoping Fan

**Affiliations:** 1 Department of Cardiovascular Surgery, Guangdong Provincial Hospital of Chinese Medicine, the Second Affiliated Hospital of Guangzhou University of Chinese Medicine, the Second Clinical College of Guangzhou University of Chinese Medicine, Guangzhou 510120, Guangdong, China.; 2 State Key Laboratory of Dampness Syndrome of Chinese Medicine, Guangzhou 510120, Guangdong, China.; 3 Guangdong Provincial Key Laboratory of TCM Emergency Research, Guangzhou 510120, Guangdong, China.; 4 Guangzhou Women and Children's Medical Center, Guangzhou Medical University, Guangzhou, 510623, Guangdong, China.; 5 Guang'anmen Hospital of Chinese Academy of Traditional Chinese Medicine, Beijing, China.; 6 Department of Clinical Laboratory Medicine, The First Medical Centre, Medical School of Chinese People's Liberation Army, Beijing, China.; 7 Department of Cardiology, Beijing Anzhen Hospital, Capital Medical University, Beijing, 100029, China.; 8 State Key Laboratory of Traditional Chinese Medicine Syndrome, Guangdong Provincial Hospital of Chinese Medicine, Guangzhou University of Chinese Medicine, Guangzhou, China.

**Keywords:** DNA-PKcs, MOTS-c, JNK, profilin, lamellipodia, endothelial barrier, myocardial microvascular injury

## Abstract

**Rationale:** The DNA-dependent protein kinase catalytic subunit (DNA-PKcs) promotes pathological mitochondrial fission during septic acute kidney injury. The mitochondrial open reading frame of the 12S rRNA type-c (MOTS-c) is a mitochondria-derived peptide that exhibits anti-inflammatory properties during cardiovascular illnesses. We explored whether endotoxemia-induced myocardial microvascular injury involved DNA-PKcs and MOTS-c dysregulation.

**Methods:** To induce endotoxemia *in vivo,* endothelial cell-specific* DNA-PKcs-*knockout mice were injected intraperitoneally with a single dose of lipopolysaccharide (10 mg/kg) and evaluated after 72 h.

**Results:** Lipopolysaccharide exposure increased DNA-PKcs activity in cardiac microvascular endothelial cells, while pharmacological inhibition or endothelial cell-specific genetic ablation of *DNA-PKcs* reduced lipopolysaccharide-induced myocardial microvascular dysfunction. Proteomic analyses showed that endothelial *DNA-PKcs* ablation primarily altered mitochondrial protein expression. Verification assays confirmed that DNA-PKcs drastically repressed *MOTS-c* transcription by inducing mtDNA breaks via pathological mitochondrial fission. Inhibiting *MOTS-c* neutralized the endothelial protective effects of *DNA-PKcs* ablation, whereas MOTS-c supplementation enhanced endothelial barrier function and myocardial microvascular homeostasis under lipopolysaccharide stress. In molecular studies, MOTS-c downregulation disinhibited c-Jun N-terminal kinase (JNK), allowing JNK to phosphorylate profilin-S173. Inhibiting JNK or transfecting cells with a profilin phosphorylation-defective mutant improved endothelial barrier function by preventing F-actin depolymerization and lamellipodial degradation following lipopolysaccharide treatment.

**Conclusions:** DNA-PKcs inactivation during endotoxemia could be a worthwhile therapeutic strategy to restore MOTS-c expression, prevent JNK-induced profilin phosphorylation, improve F-actin polymerization, and enhance lamellipodial integrity, ultimately ameliorating endothelial barrier function and reducing myocardial microvascular injury.

## Introduction

Endotoxemic endothelial damage and microvascular disorders can occur following endotoxemia, i.e., the presence of bacterial endotoxins such as lipopolysaccharides in the bloodstream. The endothelium is critical for maintaining heart performance through various physiological adaptive responses. Its damage can increase vascular permeability, reduce vasodilation, activate the coagulation cascade, promote thrombosis formation, impair blood flow, and reduce oxygen delivery to the myocardium, ultimately causing acute heart depression, known as endotoxemic cardiomyopathy 1, 2; cardiac microvascular injury is a determinant of the heart output following endotoxemia. Unfortunately, the typical treatment during endotoxemia is fluid resuscitation and vasopressor support, which can further impair the endothelial integrity 3-5. Thus, elucidating the molecular pathways underlying endothelial damage and myocardial microvascular disorder during endotoxemia is essential for developing new therapies.

The endothelial barrier is a selectively permeable membrane that separates the blood from the underlying tissue and regulates the exchange of nutrients, gases, and other molecules 6. Disruption of the endothelial barrier can increase its permeability, resulting in tissue edema and inflammation 7. Profilin has been shown to cooperate with actin and other cytoskeletal proteins to alter the structure and function of endothelial cells (ECs) 8 by forming actin stress fibers and adherens junctions, which are key components of the endothelial barrier 9, 10. Dysregulation of profilin expression, especially via phosphorylation, promotes microvascular wall disassembly and microvessel hyperpermeability 11, thereby allowing the permeation of pro-inflammatory cytokines, neutrophils, coagulation factors, and damage-associated molecular patterns. Profilin phosphorylation-induced barrier dysfunction and endothelial hyperpermeability successively trigger osmotic pressure changes, tissue edema, capillary compression, and heart ischemia 12, 13. Moreover, endothelial barrier damage promotes the interaction between circulatory neutrophils and ECs, thus shifting ECs from a quiescent status to a pro-inflammatory phenotype 14, 15 and augmenting the endotoxemia-initiated inflammatory response. Although profilin phosphorylation has been widely reported to impair endothelial barrier function, less is known about how endotoxemia alters the phosphorylation status of profilin.

The mitochondrial open reading frame of the 12S rRNA type-c (MOTS-c) is a mitochondria-derived peptide encoded in the 12S rRNA region of the mitochondrial genome 16. Increased MOTS-c expression has been associated with a reduced inflammatory response in heart tissue following pressure overload 17. MOTS-c has also exhibited anti-inflammatory properties in a mouse dextran sulfate sodium-induced colitis model 18, possibly by suppressing the c-Jun N-terminal kinase (JNK) pathway 19 and stimulating the adenosine monophosphate-activated protein kinase pathway 20. MOTS-c downregulation has been associated with JNK activation 21. However, the involvement of MOTS-c in endotoxemia-induced microvascular disorder has not been fully investigated.

Our studies and other investigations have identified the DNA-dependent protein kinase catalytic subunit (DNA-PKcs) as a pathological factor for lipopolysaccharide-induced dysfunction of multiple organs, including the heart, liver, and kidneys 22, 23. As a DNA-repair protein, DNA-PKcs amplifies immune or inflammatory signals by sensing nucleic acid damage 24. However, the downstream effects of DNA-PKcs activation extend beyond DNA repair; DNA-PKcs activation is associated with diverse events such as mitochondrial dysfunction 25, cytoskeletal disorganization 26, Golgi fragmentation 27, and heat-shock-protein phosphorylation 28. During lipopolysaccharide-stimulated endotoxemia, activated DNA-PKcs induced mitochondrial fission via mitochondrial fission 1 protein (Fis1) 23, suggesting that DNA-PKcs activation is associated with mitochondrial abnormalities. Since *MOTS-c* downregulation often results from mitochondrial dysfunction 29, we wondered whether DNA-PKcs destabilizes *MOTS-c*. In the present study, we evaluated the effects of endotoxemia on DNA-PKcs activation, MOTC-s expression, and JNK pathway activation, along with their influence on profilin phosphorylation, endothelial function, and myocardial microvascular integrity.

## Materials and Methods

### Mice

Homozygous *DNA-PKcs^f/f^* mice were generated as previously described 30. *Tie2^Cre^* transgenic mice (B6.Cg-Tg(Tek-cre)1Ywa/J, stock no: 008863) were obtained from The Jackson Laboratory (Bar Harbor, ME, USA). *DNA-PKcs^f/f^* mice were bred with *Tie2^Cre^* mice to generate EC-specific *DNA-PKcs-*knockout (*DNA-PKcs^f/f^*/*Tie2^Cre^*) mice. *DNA-PKcs^f/f^* mice were used as the control group for *DNA-PKcs^f/f^*/*Tie2^Cre^* mice*.* All mouse strains had a C57BL/6J background and were housed under specific pathogen-free conditions with a 12 h/12 h light/dark cycle under controlled temperature and humidity. Food and water were provided *ad libitum*.

### Human sample collection and ethical approval

All patients signed informed consent forms prior to their inclusion. Blood samples were collected from patients with (n = 66) and without (n = 174) septic cardiomyopathy in intensive care units. Septic cardiomyopathy was identified based on an acutely depressed LVEF with ventricular dilation occurring during sepsis 31. Patient characteristics are given in Table S1. CD34^+^ ECs and EPCs were collected with the assistance of magnetic polystyrene beads coated with a monoclonal antibody in accordance with a previous study 32.

### Experimental model

Eight-week-old male mice were injected intraperitoneally with a single dose of lipopolysaccharide (10 mg/kg) to induce endotoxemia, and were evaluated after 72 h, following a previously outlined method 23. Phosphate-buffered saline (PBS) was used as the vehicle. MOTS-c (15 mg/kg, Phoenix Pharmaceuticals, Inc., cat. no.: 038-48) was administered daily via intraperitoneal injection for seven days before the mice were subjected to lipopolysaccharide-induced endotoxemia. DNA-PKcs activity was inhibited via intraperitoneal injection of NU7441 (2 mg/kg, Selleck, cat. no.: S7409) three days before lipopolysaccharide treatment.

### Histological, immunohistochemical, and immunofluorescence staining

Heart tissues were fixed with 4 % paraformaldehyde for 24 h, embedded in paraffin using standard procedures, and then cut into 5-μm-thick sections. The tissue sections were stained with H&E for structural evaluation, dehydrated for immunohistochemical and immunofluorescence staining, and then antigens were retrieved through an overnight incubation at 4 °C with the primary antibodies listed in Table S2. Subsequently, the sections were incubated with horseradish peroxidase-conjugated or fluorescence-conjugated secondary antibodies (1:500; Jackson Laboratory) at 20 °C for 1 h.

### qPCR, mtDNA strand-break detection, and circulating DNA purification

Total RNA was extracted from heart tissues and cell samples using an RNeasy Mini Kit (Qiagen, Hilden, Germany), and 800 ng of RNA was reverse-transcribed into cDNA using a PrimeScript RT Reagent Kit (Takara, Tokyo, Japan) according to the manufacturer's instructions. Relative gene expression was measured using a SYBR Green PCR Kit (Takara) with *β-Actin* as an internal control. All qPCR primers are listed in Table S3. For the detection of *MOTS-c* transcription, 50 ng of fractioned RNA was isolated and then converted to cDNA using a TaqMan® microRNA RT kit (#4366596; Life Technologies, Carlsbad, CA, USA). Subsequently, a specific TaqMan® Small RNA Assay (Life Technologies) for *MOTS-c* primers (designed using the Custom TaqMan® Small RNA Assay Design Tool against the *MOTS-c* mtDNA sequence) was used to analyze *MOTS-c* transcription per the manufacturer's instructions.

Strand breaks in mtDNA were analyzed based on our previous studies. In brief, a mitochondrial suspension was isolated and then centrifuged at 15000 x *g* at 4 °C for 20 min. The supernatant was removed, and the sediment was mixed with 400 µL of a solution containing 0.25 mM inositol, 10 mM Na_3_PO_4_ and 1 mM MgCl_2_ (pH 7.2) at 4 °C for 30 min. The remaining steps were conducted according to the fluorometric analysis of DNA unwinding ('FADU') methods reported by Birnboim and Jevcak 33, and the proportion of mtDNA strand breaks (normalized to the control group) was recorded.

Circulating DNA was extracted from 250 μL of plasma using the ENZA Circulating DNA Kit (Omega Bio-Tek, Norcross, GA, USA) following the manufacturer's instructions. The copy number of mtDNA (cytochrome c oxidase 1, mt-COI) was quantified by quantitative PCR with SYBR Green PCR Mix (GeneCopoeia, Shanghai, China) and normalized to nuclear DNA (18s RNA) levels. PCR reactions were performed in a final volume of 20 μL, comprising 0.2 μM of each forward and reverse primer, 20 ng of DNA sample, and 10 μL of SYBR Green PCR Mix. Amplification was carried out using an Applied Biosystems 7300 Real-Time PCR machine (Life Technologies, MA, USA) with the following thermal profile: initial denaturation at 95 °C for 10 min, followed by 40 cycles of denaturation at 95 °C for 15 s, annealing at 60 °C for 20 s, and extension at 72 °C for 20 s. The threshold cycle (Ct) values were obtained from duplicate samples and averaged. The levels of the target gene were determined using the ΔΔCt method and expressed relative to the control group.

### Cellular immunofluorescence staining and mitochondrial membrane potential

Cells were seeded onto gelatin-coated cytospin slides, air-dried, and then fixed in paraformaldehyde at 20 °C for 30 min. After being washed with PBS, the cells were permeabilized with 0.3% Triton X-100 for 15 min, blocked with 10% bovine serum albumin at 20 °C for 1 h, incubated with primary antibodies at 4 °C overnight, and then treated with fluorescence-conjugated secondary antibodies for 1 h. Nuclei were stained with 4',6-diamidino-2-phenylindole (DAPI). The antibodies used for immunofluorescence are shown in Table S2. The mitochondrial membrane potential was determined using JC-1 as per a previously described method 34.

### Western blot analysis

For Western blot analysis of heart tissues or cultured cell samples, a previously described protocol was followed. Briefly, the samples were lysed in ice-cold radioimmunoprecipitation assay lysis buffer containing 10 mM Tris-Cl (pH 8.0), 1 mM ethylenediamine tetraacetic acid, 1% Triton X-100, 0.1% sodium deoxycholate, 0.1% sodium dodecyl sulfate, 150 mM NaCl, 1 mM phenylmethylsulfonyl fluoride, and 0.02 mg/mL each of aprotinin, leupeptin, and pepstatin. After sonication, the lysates were clarified by centrifugation. Protein quantification was performed using a Bradford analysis, and 50-100 mg of proteins per lane were resolved by sodium dodecyl sulfate polyacrylamide gel electrophoresis. The proteins were subsequently transferred to a polyvinylidene difluoride membrane and probed with the antibodies listed in Table S2.

### FITC-dextran clearance assay and TER experiment

For the assessment of EC permeability, cells were treated with FITC-dextran (1 mg/mL, cat. no.: 46944-100MG-F, Sigma) for 2 h. The cells were then washed with PBS, and the residual FITC-dextran was observed using a fluorescent plate reader (Bio-Rad, USA). An *in vitro* vascular permeability assay kit (ECM640, Millipore, USA) was used to measure the ionic conductance of ECs seeded onto collagen-coated inserts at a density of 100,000 cells/insert, with the assistance of an electrical endothelial resistance system (Millipore), as per a previously described method 35.

### Echocardiogram

Mice were placed under light anesthesia with 1-2% isoflurane, and two-dimensional targeted M-mode images were obtained from the short axis view at the papillary muscle level using a Vevo 2100 ultrasonography system (VisualSonics, Toronto, Canada), as previously described. The left ventricular diastolic/systolic diameter, LVEF, and fractional shortening (FS) were assessed. The left ventricular FS was calculated as %FS = [(diastolic left ventricular diameter - systolic left ventricular diameter)/diastolic left ventricular diameter] × 100.

### ELISA

The levels of TnT (Mouse Troponin T (TNT) ELISA Kit, cat. no.: abx585262, Abbexa Ltd.), CK-MB (Mouse CK-MB/Creatine Kinase-MB (Sandwich ELISA) ELISA Kit, cat. no.: LS-F5745, LifeSpan BioSciences, Inc.), BNP (Mouse BNP ELISA Kit, cat. no.: MBS2510603, MyBioSource, Inc.), LDH (Mouse Lactate Dehydrogenase (LDH) ELISA Kit, cat. no.: abx154299, Abbexa Ltd.), DNA-PKcs (Mouse PRKDC/DNA-PKcs (Sandwich ELISA) ELISA Kit, cat. no.: LS-F8115, LifeSpan BioSciences, Inc.) and MOTS-c (Mouse Mitochondrial Open Reading Frame of the 12S rRNA-c (MOTS-c) ELISA Kit, cat. no.: MBS2090467, MyBioSource, Inc.) were detected using ELISA kits according to the manufacturers' instructions.

### Neonatal cardiac microvascular endothelial cell (CMEC) isolation

Two-week-old *DNA-PKcs^f/f^* and *DNA-PKcs^f/f^*/*Tie2^Cre^* mice were anesthetized with 1% sodium, and the left ventricle was collected from each mouse under sterile conditions. The tissues were immersed in 75% ethanol for 15 s to devitalize the epicardial and endocardial cells and then digested with 0.2% (w/v) collagenase type I (Gibco, USA) for 10 min and 0.25% (w/v) trypsin (Hyclone, USA) for 5 min at 37 °C in a shaking bath, a method based on our previous studies 35, 36. The cells were filtered with 70-μm Cell Strainers (cat. no.: 15-1070, Biologix) to remove debris. Then, the cells were washed twice with ice-cold Dulbecco's Modified Eagle's Medium, centrifuged at 200 x *g* for 3 min, and seeded at 1x 10^6^ cells per 60-mm dish. The dishes were pre-coated with collagen (cat. no.: C7661, Sigma).

### Cell lines and treatment

Human coronary arterial endothelial cells (HCAECs) were purchased from the Shanghai Cell Collection (Shanghai, China). All cells were cultured in the recommended medium supplemented with 10% fetal bovine serum (Biological Industries, USA) and 1% penicillin-streptomycin (Gibco, USA) at 37 °C with 5% CO_2_. All cell lines were authenticated using short tandem repeat profiling and were routinely tested for mycoplasma. HCAECs were treated with 10 μg/mL lipopolysaccharide for 24 h. HCAECs were treated with NU7441 (1 μM, Selleck, cat. no.: S2638) for 2 h before lipopolysaccharide treatment. HCAECs were treated with MOTS-c (10 µM, Eurogentec, cat. no.: AS-65587) or the vehicle (PBS) 24 h before lipopolysaccharide administration. HCAECs were treated with SCH772984 (5 μM, Selleck, cat. no.: S7101) or LM22B-10 (10 μM, Selleck, cat. no.: S6760) to inhibit or activate the ERK1/2 pathway, respectively. HCAECs were treated with anisomycin (2 μM, Selleck, cat. no.: S7409) or SP600125 (10 μM, Selleck, cat. no.: S7409) to activate or inactivate the JNK pathway, respectively. HCAECs were treated with ERK5-IN-1 (10 μM, Selleck, cat. no.: S7334) or SB202190 (5 μM, Selleck, cat. no.: S1077) to inhibit ERK5 and p38, respectively. HCAECs were treated with Jasplakinolide (20 μM, Santa Cruz Biotechnology, cat. no.: sc-202191) or Cytochalasin D (15 μM, Santa Cruz Biotechnology, cat. no.: sc-201442) to induce or suppress F-actin polymerization, respectively. HCAECs were treated with 10 nM Mdivi-1 (Selleck, cat. no.: S7162) or 30 μM FCCP (Selleck, cat. no.: S8276) to inhibit or activate mitochondrial fission, respectively. HCAECs were treated with actinonin (20 nM, MedChemExpress, cat. no.: HY-113952) to inhibit the transcription of mitochondrial 12S rRNA, a method based on a previous study 37.

### Plasmids and lentiviruses

The pcDNA3.1 (vector) and pcDNA3-DNA-PKcs (pIRF8) plasmids were constructed by GenScript (Nanjing, China). *DNA-PKcs* shRNA (human) Lentiviral Particles containing four target-specific constructs (cat. no.: #sc-35200-V) and scrambled shRNA Lentiviral Particles (cat. no.: sc-108080) were purchased from Santa Cruz Biotechnology. *DNA-PKcs*-knockdown (sh-DNA-PKcs) and control (sh-scramble) cell lines were generated via stable infection of cells with the corresponding lentiviral particles. Stably transduced cells were selected using puromycin (2-4 μg/mL) in the culture medium.

### Profilin mutant plasmid transfection

Profilin^S273^ site-mutation plasmids containing a phosphorylation-simulating mutation (profilin^S273D^) or a phosphorylation-defective mutation (profilin^S273A^) were constructed by Shandong Vigene Biosciences. The expression plasmids were transfected into HCAECs with a Lipofectamine 3000 reagent (Invitrogen, USA) to simulate profilin's phosphorylation or non-phosphorylation status, respectively.

### Co-immunoprecipitation assay

HCAECs were transfected with profilin mutation plasmids for 12 h, followed by lipopolysaccharide treatment. The cells were then washed with cold PBS and lysed with a radioimmunoprecipitation assay buffer. After the protein concentration was adjusted to 500 μg/mL, a primary antibody, or IgG as the negative control, was added, and the samples were incubated at 4 ℃ with rotation overnight. Protein G magnetic beads were added for 12 h, followed by three washes with cold Tris buffer (50 mM, pH 7.4). The pulldown proteins were directly resuspended with loading buffer and separated via sodium dodecyl sulfate polyacrylamide gel electrophoresis. The proteins were transferred to polyvinylidene difluoride membranes, which were then incubated with the corresponding primary antibodies, followed by secondary antibodies. The bands were visualized using a chemiluminescence assay.

### Proteomic analysis via mass spectrometry

After reaching 70% confluence, CMECs (n = 3 from each cell type) were collected and washed with PBS. The cells were then pelleted and stored at -80 °C for subsequent mass spectrometry analysis. Total proteins for proteomic analysis were extracted using a mixture of urea, thiourea, and 4% 3-[(3-cholamidopropyl)dimethylammonio]-1-propanesulfonate. The samples were incubated at room temperature with agitation for 30 minutes, followed by digestion using a modified filter-aided sample preparation protocol. Trypsin was added at a trypsin:protein ratio of 1:10, and the mixture was incubated overnight at 37 °C. The digested samples were dried using an RVC2-25 rotational vacuum concentrator (Martin Christ, Germany) and resuspended in 0.1% formic acid.

For mass spectrometry analysis, a hybrid trapped ion mobility spectrometry - quadrupole time of flight mass spectrometer (timsTOF Pro with PASEF, Bruker Daltonics) coupled to a nanoElute liquid chromatograph (Bruker) was used. The samples were loaded directly into a 15-cm Bruker nanoElute FIFTEEN C18 analytical column and resolved at a flow rate of 400 nL/min with a 30-minute gradient. The column was maintained at 50 °C throughout the analysis. Protein identification and quantification were performed using PEAKS software (Bioinformatics Solutions, Inc.). Searches were conducted against a database of human entries (Uniprot/Swiss-Prot), with precursor and fragment tolerances set at 20 ppm and 0.05 Da, respectively. To normalize peptide quantification data, non-conflicting peptide abundances were exported from PEAKS and processed with the proBatch package in R. Finally, normalized data were compiled at the protein level and log_2_-transformed. For the differential analysis using IBM SPSS v22, the Shapiro-Wilk normality test was performed to evaluate expression normality, and then a parametric T-test or non-parametric Mann-Whitney test was conducted to detect statistically significant differences. The freely available web server Heatmapper was used to graph protein expression heatmaps. For the classification of differentially expressed proteins, functional enrichment analyses for biological processes or pathways were performed using the FunRich database.

### GSEA

A ranked list of differentially expressed genes was generated based on log_2_fold-change values. Subsequently, a GSEA was performed using the GSEA function of the R/Bioconductor clusterProfiler package. The enrichment score (ES) was calculated by applying the weighted Kolmogorov-Smirnov statistic to a running sum of the ranked list. The ES was further normalized to account for the size of each gene set. Statistical significance was determined based on a false discovery rate < 0.05.

### Statistical analysis

All data are presented as the mean ± standard error of at least three biological replicates per group. Data were analyzed using Student's *t-*test for two groups, or one-way analysis of variance for multiple groups. Survival curves were compared using the log-rank (Mantel-Cox) test. All data were analyzed using GraphPad Prism 7 software (GraphPad Software, San Diego, CA, USA). P-values < 0.05 were considered statistically significant.

## Results

### Lipopolysaccharide exposure upregulates DNA-PKcs in CMECs

**Figure 1:**
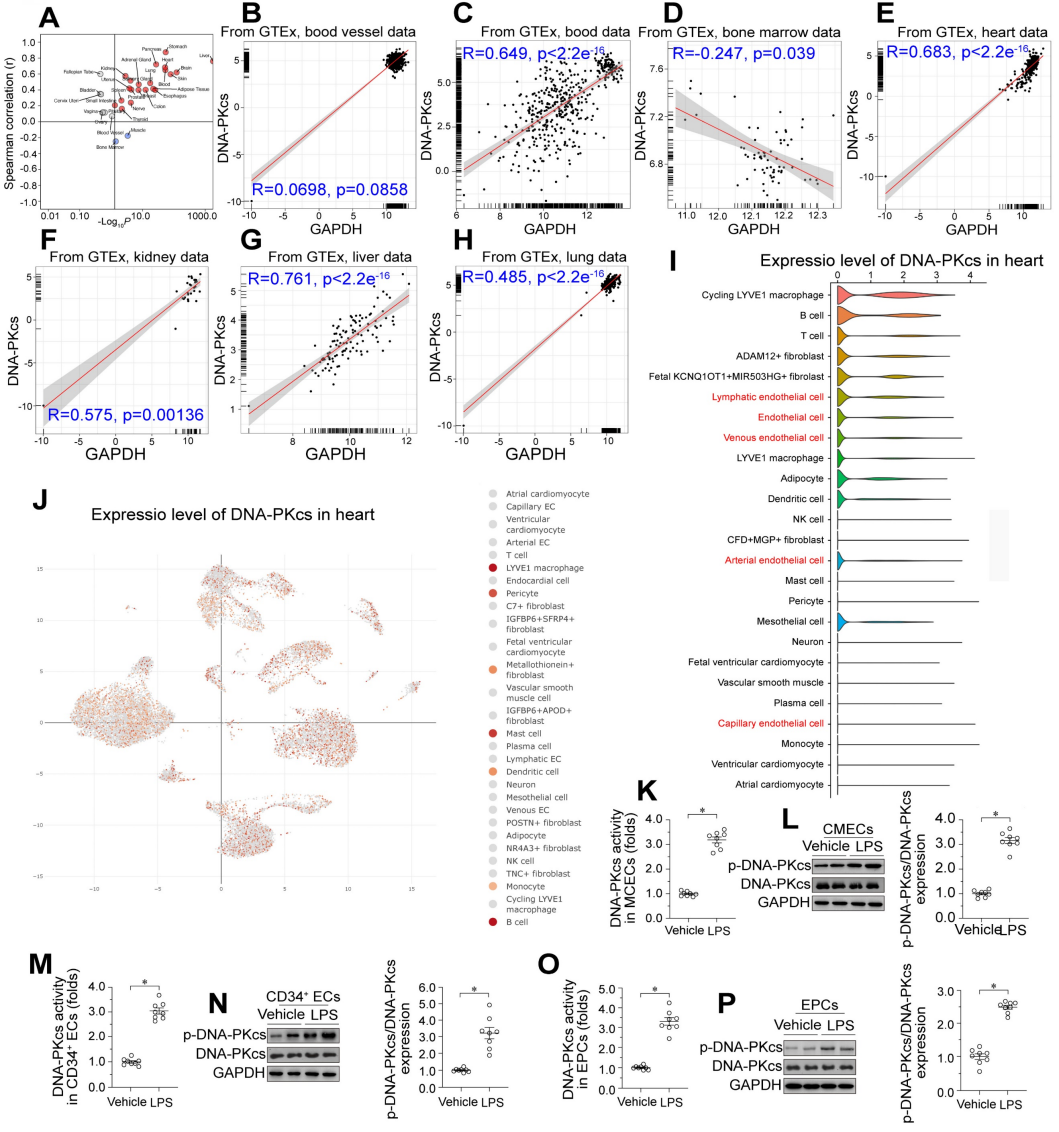
** Lipopolysaccharide upregulates DNA-PKcs in cardiac microvascular ECs, leading to vascular dysfunction. A-H.** Genotype-Tissue Expression analysis results for *DNA-PKcs* in the heart, liver, kidneys, blood vessels, blood, bone marrow and lungs. **I, J.** *DNA-PKcs* expression in different cell types from the heart, determined using single-cell sequencing analysis. **K.** An ELISA kit was used to evaluate DNA-PKcs activity in CMECs isolated from WT mice with or without lipopolysaccharide treatment.** L.** Proteins were extracted from CMECs isolated from WT mice with or without lipopolysaccharide treatment, and phosphorylated DNA-PKcs levels were determined using Western blotting. **M.** CD34^+^ ECs were isolated from septic patients' blood samples using flow cytometry, and an ELISA kit was used to analyze DNA-PKcs activity in these cells (40,000 cells/well). **N.** CD34^+^ ECs were isolated from septic patients' blood samples using flow cytometry, and Western blotting was used to detect DNA-PKcs phosphorylation in these cells (100,000 cells/group). **O.** EPCs were isolated from septic patients' blood samples using flow cytometry, and an ELISA kit was used to analyze DNA-PKcs activity in these cells (100,000 cells/well). **P.** EPCs were isolated from septic patients' blood samples using flow cytometry, and Western blotting was used to detect DNA-PKcs phosphorylation in these cells (500,000 cells/group). Experiments were repeated at least three times, and the data are shown as mean ± SEM (N = 8 mice or eight independent cell isolations per group). * p < 0.05.

We used the Genotype-Tissue Expression database of RNA sequencing (RNA-seq) data to investigate *DNA-PKcs* expression across various human tissues. *DNA-PKcs* was predominantly expressed in liver tissue, followed by the heart, blood, kidneys, lungs, blood vessels, and bone marrow in decreasing order (Figure 1A-H). To determine which cell type was the primary source of *DNA-PKcs* in heart tissue, we performed single-cell analyses using the Deeply Integrated Human Single-Cell Omics (DISCO) database (https://www.immunesinglecell.org/), which contains single-cell RNA-seq data from 198 heart samples from healthy donors 38. The results indicated that *DNA-PKcs* was abundant in myocardial macrophages and other immunocytes such as B cells and T cells (Figure 1I-J). *DNA-PKcs* was also detected in cardiac ECs such as lymphatic ECs, arterial ECs, venous ECs, and capillary ECs (Figure 1I-J). Interestingly, there was little *DNA-PKcs* expression in cardiomyocytes and smooth muscle (Figure 1I-J). Since ECs, cardiomyocytes, and smooth muscle are the primary components of heart tissue, we concluded that *DNA-PKcs* expression in the heart is largely determined by *DNA-PKcs* levels in ECs.

To assess the effects of endotoxemia on DNA-PKcs levels in cardiac microvascular ECs, we isolated neonatal CMECs from wild-type (WT) mice and treated them with lipopolysaccharides. ELISA and Western blotting analyses showed that DNA-PKcs activity and phosphorylation, respectively, were significantly elevated in CMECs following lipopolysaccharide treatment (Figure 1K-L). To evaluate the clinical relevance of DNA-PKcs activation in endotoxemia-induced myocardial microvascular dysfunction, we isolated human circulating CD34^+^ ECs from septic patients and healthy subjects, as these cells participate in the angiogenesis and repair of the myocardial microcirculation during endotoxemia. We also collected endothelial progenitor cells (EPCs) from the peripheral blood of these subjects. ELISAs and Western blotting revealed that DNA-PKcs activity and phosphorylation, respectively, were significantly elevated in circulating CD34^+^ ECs (Figure 1M-N) and EPCs (Figure 1O-P) from septic patients compared with healthy subjects. Moreover, higher DNA-PKcs activity levels in EPCs were associated with higher Acute Physiology and Chronic Health Evaluation (APACHE) II scores, higher Sequential Organ Failure Assessment (SOFA) scores, lower left ventricular ejection fraction (LVEF) values, and higher lactic acid levels (Table S4). In addition, we used flow-mediated dilation (FMD), endothelial peripheral artery tonometry reactive hyperemia index (endo-PAT RHI), carotid-to-femoral pulse wave velocity (CF-PWV), cardio-ankle vascular index (CAVI) and ankle-brachial index (ABI) measurements to determine the clinical association of higher EPC DNA-PKcs activity with cardiac endothelial dysfunction (Table S4). The results demonstrated that increased DNA-PKcs activity was associated with reduced endothelial function.

### *DNA-PKcs* deletion attenuates endotoxemia-induced myocardial microvascular endothelial injury

**Figure 2:**
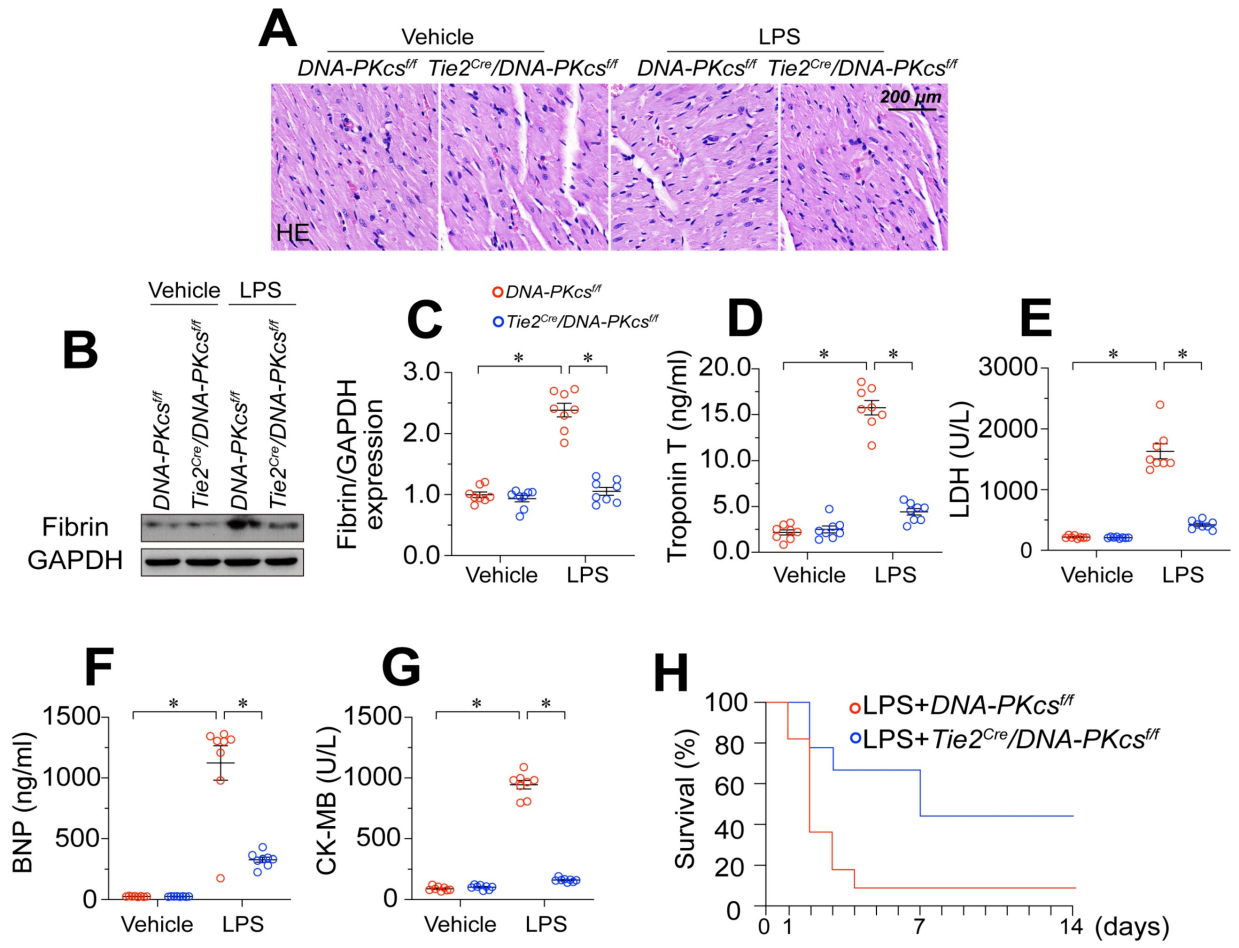
***DNA-PKcs* deletion attenuates endotoxemia-induced myocardial microvascular endothelial injury.** To induce endotoxemia *in vivo, DNA-PKcs^f/f^* and *DNA-PKcs^f/f^*/*Tie2^Cre^* male mice (eight weeks old) were injected intraperitoneally with a single dose of lipopolysaccharide (10 mg/kg) and evaluated after 72 h. **A.** H&E staining of erythrocyte aggregation in microvessels after lipopolysaccharide treatment. **B, C.** Proteins were extracted from CMECs isolated from mice with or without lipopolysaccharide treatment, and fibrin expression was determined using Western blotting. **D-G.** ELISA kit analysis of cardiac injury biomarkers (serum TnT, CK-MB, LDH, and BNP). **H.** Survival times of different mice in the presence or absence of lipopolysaccharides. Experiments were repeated at least three times and the data are shown as mean ± SEM (N = 8 mice or eight independent cell isolations per group). * p < 0.05.

To determine whether increased DNA-PKcs activity influenced the pathology of endotoxemia-induced myocardial microvascular endothelial injury, we crossed *DNA-PKcs^f/f^* mice with *Tie^Cre^* mice to produce EC-specific *DNA-PKcs*-knockout (*DNA-PKcs^f/f^*/*Tie^Cre^*) mice. These mice, along with *DNA-PKcs^f/f^* control mice, were then treated with lipopolysaccharides or the vehicle. Hematoxylin and eosin (H&E) staining revealed erythrocyte aggregation in microvessels following lipopolysaccharide treatment in *DNA-PKcs^f/f^* control mice (Figure 2A). Western blotting of heart tissues demonstrated that lipopolysaccharide stress induced fibrin expression in* DNA-PKcs^f/f^* control mice (Figure 2B-C), indicating that coagulation reactions had increased. However, ablation of *DNA-PKcs* in ECs reduced erythrocyte accumulation (Figure 2A) and fibrin deposition (Figure 2B-C).

Given the importance of the microvasculature for heart function, we asked whether the protective effects of *DNA-PKcs* deficiency on coronary integrity were associated with improved heart function. ELISAs demonstrated that lipopolysaccharides upregulated cardiac injury biomarkers such as serum troponin T (TnT), creatine kinase (CK)-MB, natriuretic peptide B (BNP) and lactate dehydrogenase (LDH) in control *DNA-PKcs^f/f^* mice, but not in *DNA-PKcs^f/f^/Tie^Cre^* mice (Figure 2D-G). We also performed echocardiography, which indicated that heart function was disrupted by lipopolysaccharides in the control *DNA-PKcs^f/f^* mice but partially normalized in lipopolysaccharide-treated *DNA-PKcs^f/f^/Tie^Cre^* mice (Table S5). Due to their improved microvascular structure and myocardial function, *DNA-PKcs^f/f^/Tie^Cre^* mice also survived 40% longer than control* DNA-PKcs^f/f^* mice in the presence of lipopolysaccharide stress (Figure 2H).

In addition to genetically modifying *DNA-PKcs in vivo*, we also used a pharmacological inhibitor of DNA-PKcs (NU7441) to exclude the influence of *DNA-PKcs* deficiency on the phenotypic alterations during endotoxemia. Consistent with the above results, the pharmacological blockade of DNA-PKcs reduced erythrocyte accumulation (Figure S1A), and prevented coagulation activation (Figure S1B-C), ultimately suppressing myocardial injury (Figure S1D-G), sustaining heart function (Table S6) and extending the survival time (Figure S1H) in a murine lipopolysaccharide-induced endotoxemia model. Interestingly, compared with the genetic modification strategy, pharmacological suppression of DNA-PKcs may have further protected the microvascular integrity and myocardial performance, possibly due to the dual effects of NU7441 on ECs and cardiomyocytes. Therefore, to observe the effects of DNA-PKcs inactivation specifically on EC pathology and microvascular injury during endotoxemia, we used the genetically modified mice for subsequent functional experiments. These results illustrated that endotoxemia-induced myocardial microvascular damage depends on DNA-PKcs activation.

### *DNA-PKcs* depletion preserves cardiac microvascular endothelial barrier function during endotoxemia

**Figure 3:**
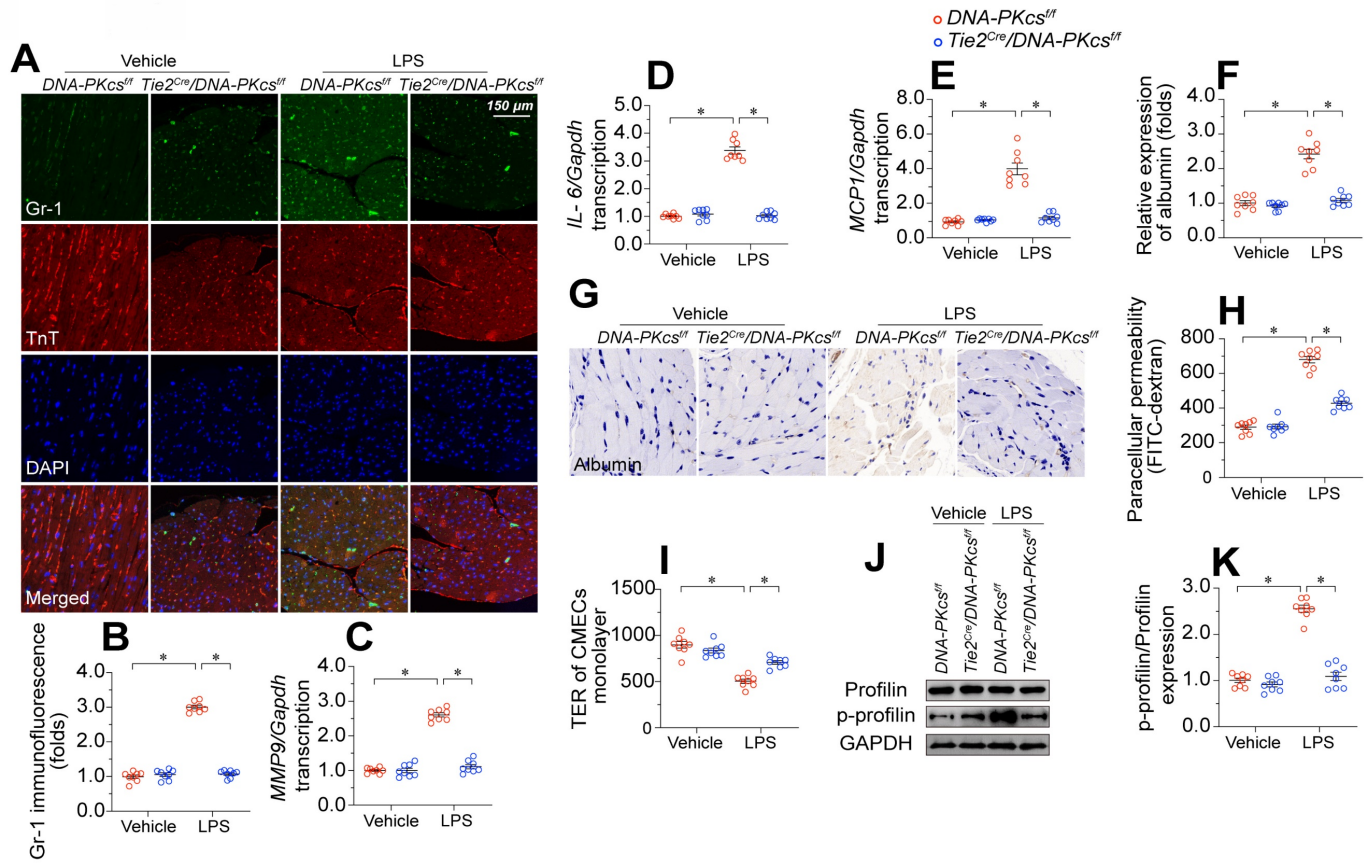
***DNA-PKcs* depletion protects cardiac microvascular endothelial barrier function during endotoxemia.** To induce endotoxemia *in vivo, DNA-PKcs^f/f^* and *DNA-PKcs^f/f^*/*Tie2^Cre^* male mice (eight weeks old) were injected intraperitoneally with a single dose of lipopolysaccharide (10 mg/kg) and evaluated after 72 h. HCAECs were transfected with sh-DNA-PKcs or sh-scramble before treatment with 10 μg/mL of lipopolysaccharides. **A, B.** Immunofluorescence staining of GR-1-positive neutrophils in the myocardium. **C-E.** RNA was isolated from heart tissue, and qPCR was used to determine the transcription of *IL-6*, *MCP1,* and *MMP9*. **F, G.** Immunohistochemical analysis of albumin in the myocardium following lipopolysaccharide stress. **H.** CMECs were isolated from *DNA-PKcs^f/f^* and *DNA-PKcs^f/f^*/*Tie2^Cre^* mice and then treated with lipopolysaccharides. Then, a FITC clearance assay was used to analyze EC permeability *in vitro*. **I.** A TER assay was used to observe changes in the endothelial barrier integrity in the presence of lipopolysaccharides. **J, K.** Proteins were isolated from CMECs with or without lipopolysaccharide treatment, and total or phosphorylated profilin levels were determined using Western blotting. Experiments were repeated at least three times and the data are shown as mean ± SEM (N = 8 mice or eight independent cell isolations per group). * p < 0.05.

EC barrier dysfunction has been regarded as an early sign of myocardial microvascular injury and is characterized by neutrophil invasion, pro-inflammatory cytokine overexpression, and tissue edema. Double-immunofluorescence staining showed that lipopolysaccharide exposure increased GR-1-positive neutrophil localization within the myocardium in control *DNA-PKcs^f/f^* mice but not in *DNA-PKcs^f/f^/Tie^Cre^* mice (Figure 3A-B). In addition, pro-inflammatory cytokines such as interleukin 6 (*IL-6*), monocyte chemoattractant protein 1 (*MCP1*), and matrix metalloproteinase 9 (*MMP9*) were significantly upregulated upon lipopolysaccharide treatment in control *DNA-PKcs^f/f^* hearts, but not in *DNA-PKcs^f/f^/Tie^Cre^* hearts (Figure 3C-E). Although albumin is exclusively detected in the microvessels under physiological conditions, immunohistochemistry revealed albumin infiltration into the myocardium following lipopolysaccharide stress (Figure 3F-G). Ablation of *DNA-PKcs* in ECs prevented the leakage of albumin from the microvasculature into the myocardium after lipopolysaccharide treatment.

To further explore the involvement of DNA-PKcs in EC barrier function, we isolated neonatal CMECs from control *DNA-PKcs^f/f^* mice and *DNA-PKcs^f/f^/Tie^Cre^* mice and treated them with lipopolysaccharides. Then, we performed fluorescein isothiocyanate (FITC)-dextran clearance and transendothelial electrical resistance (TER) assays. As a result of increased EC permeabilization, the residual FITC content was significantly elevated after lipopolysaccharide exposure in *DNA-PKcs^f/f^* mice-derived CMECs (*DNA-PKcs^f/f^* CMECs), whereas this alteration was reduced in *DNA-PKcs^f/f^/Tie^Cre^* mice-derived CMECs (*DNA-PKcs^f/f^/Tie^Cre^* CMECs) (Figure 3H). Moreover, lipopolysaccharide exposure reduced the TER values in *DNA-PKcs^f/f^* CMECs, but not in *DNA-PKcs^f/f^/Tie^Cre^* CMECs (Figure 3I). Since the TER value mainly depends on the area of adherent ECs on the culture dish, these results suggested that *DNA-PKcs* depletion attenuated lipopolysaccharide-induced EC disassociation from the dish.

Profilin is a critical structural protein that maintains the normal level of EC permeability by preserving cytoskeletal organization. Neither lipopolysaccharide treatment nor *DNA-PKcs* deletion altered the profilin protein expression in CMECs (Figure 3J-K). However, lipopolysaccharides markedly induced profilin phosphorylation in *DNA-PKcs^f/f^* CMECs, whereas *DNA-PKcs* ablation reduced it to near-normal levels (Figure 3J-K). These results suggested that lipopolysaccharide exposure disrupted the microvascular barrier by inducing DNA-PKcs-dependent profilin phosphorylation.

### Profilin phosphorylation promotes endothelial lamellipodial degradation by inducing F-actin depolymerization

**Figure 4:**
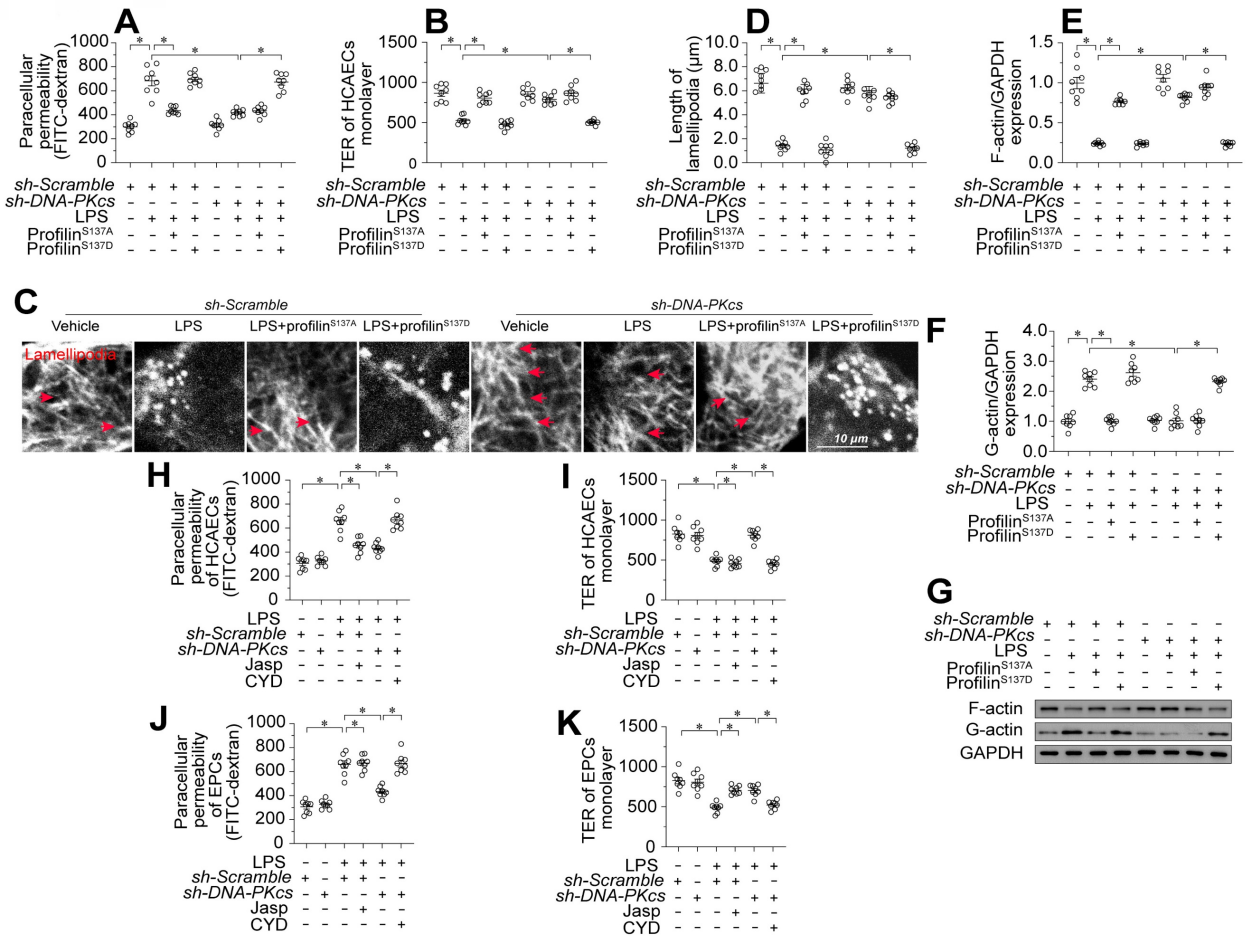
** Profilin phosphorylation promotes endothelial lamellipodial degradation by inducing F-actin depolymerization.** To induce endotoxemia *in vivo, DNA-PKcs^f/f^* and *DNA-PKcs^f/f^*/*Tie2^Cre^* male mice (eight weeks old) were injected intraperitoneally with a single dose of lipopolysaccharide (10 mg/kg) and evaluated after 72 h. HCAECs were transfected with a profilin phosphorylation-simulating mutant (profilin^S137D^) or phosphorylation-defective mutant (profilin^S137A^). **A.** A FITC clearance assay was used to analyze the permeability of HCAECs *in vitro*. **B.** A TER assay was used to observe changes in the endothelial barrier integrity in the presence of lipopolysaccharides. **C, D.** Immunofluorescence staining was used to visualize F-actin. Then, the lengths of lamellipodia were measured. **E-G.** Proteins were isolated from HCAECs with or without lipopolysaccharide treatment, and the levels of F-actin and G-actin were determined using Western blotting.** H, I.** HCAECs were treated with Jasplakinolide (20 μM) or Cytochalasin D (CYD, 15 μM) 6 h before lipopolysaccharide treatment. Then, FITC clearance and TER assays were performed to detect alterations in the endothelial barrier. **J, K.** EPCs were treated with Jasplakinolide (20 μM) or CYD (15 μM) 6 h before lipopolysaccharide treatment. Then, FITC clearance and TER assays were performed to detect alterations in the endothelial barrier. Experiments were repeated at least three times and the data are shown as mean ± SEM (N = 8 mice or eight independent cell isolations per group). * p < 0.05.

To assess whether profilin phosphorylation contributed to lipopolysaccharide-induced endothelial barrier dysfunction during endotoxemia, we transfected human coronary arterial endothelial cells (HCAECs) with either a profilin phosphorylation-simulating mutant (profilin^S137D^) or a phosphorylation-defective mutant (profilin^S137A^). Following transfection, we treated the cells with lipopolysaccharides and introduced DNA-PKcs short hairpin RNA (sh-DNA-PKcs), or scrambled shRNA (sh-scramble). Profilin^S137A^ attenuated lipopolysaccharide-induced FITC retention and TER reduction in HCAECs; however, sh-DNA-PKcs failed to improve these parameters in lipopolysaccharide-treated HCAECs transfected with profilin^S137D^ (Figure 4A-B).

Profilin phosphorylation is associated with the disassembly of lamellipodia. Immunofluorescence staining demonstrated that lipopolysaccharide noticeably reduced the number and length of endothelial lamellipodia (Figure 4C-D). Transfection with either profilin^S137A^ or sh-DNA-PKcs maintained a stable lamellipodial number and length in HCAECs following lipopolysaccharide treatment; however, in profilin^S137D^-treated HCAECs, sh-DNA-PKcs failed to prevent lipopolysaccharide-induced lamellipodial degradation (Figure 4C-D). Lamellipodia form dynamically via G-actin polymerization into F-actin. Western blotting showed that lipopolysaccharides promoted F-actin depolymerization into G-actin, whereas profilin^S137A^ reversed this change in HCAECs (Figure 4E-G). Although *DNA-PKcs* ablation maintained the abundance of F-actin in lipopolysaccharide-treated HCAECs, profilin^S137D^ seemed to nullify this effect (Figure 4E-G).

While the above results confirmed that lamellipodial formation depends on a dephosphorylated status of profilin, it remained unclear whether endothelial barrier organization relies on lamellipodial stability. To clarify this, we treated HCAECs and EPCs with Jasplakinolide (a lamellipodial polymerizer 39) or Cytochalasin D (a depolymerizer 40) before lipopolysaccharide and/or sh-DNA-PKcs treatment. Jasplakinolide inhibited lipopolysaccharide-induced FITC retention and TER reduction in both HCAECs (Figure 4H-I) and EPCs (Figure 4J-K). In contrast, although sh-DNA-PKcs transfection accelerated FITC clearance and increased the TER values in both HCAECs (Figure 4H-I) and EPCs (Figure 4J-K) after lipopolysaccharide treatment, Cytochalasin D obliterated these effects. Thus, profilin phosphorylation and consequent lamellipodial degradation seemed to be the intracellular pathological signals whereby DNA-PKcs contributed to lipopolysaccharide-induced endothelial barrier dysfunction.

### *DNA-PKcs* deletion protects mitochondrial function and structure against endotoxemia stress

**Figure 5:**
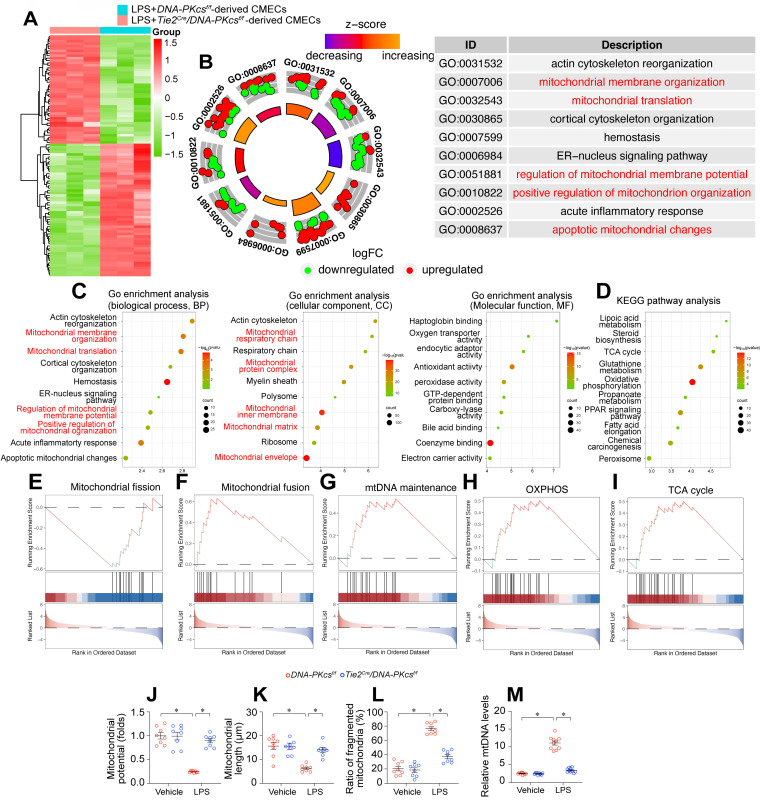
***DNA-PKcs* deletion protects mitochondria function and structure against endotoxemia stress. A.** An expression heatmap of differentially expressed proteins, showing that 490 proteins were upregulated and 595 were downregulated (1085 proteins in total) in neonatal CMECs isolated from *DNA-PKcs^f/f^/Tie2^Cre^* mice compared with control *DNA-PKcs^f/f^* mice after lipopolysaccharide treatment. N = 3 independent cell isolations per group; the experiment was performed once. **B-C.** Gene Ontology Enrichment Analysis of the 1085 differentially expressed proteins in CMECs. **D.** KEGG analysis. **E-I.** GSEA analysis of differentially expressed genes following *DNA-PKcs* deletion. **J.** JC-1 was used to determine the mitochondrial membrane potential in CMECs isolated from *DNA-PKcs^f/f^/Tie2^Cre^* mice and control *DNA-PKcs^f/f^* mice in the presence of lipopolysaccharides. Experiments were repeated at least three times and the data are shown as mean ± SEM (N = 8 mice or eight independent cell isolations per group). **K, L.** Immunofluorescence staining was used to visualize mitochondria in CMECs. Then, the average mitochondrial length and the proportion of fragmented mitochondria were measured. **M.** *DNA-PKcs^f/f^/Tie2^Cre^* mice and control *DNA-PKcs^f/f^* mice were injected with lipopolysaccharides, and plasma was collected for circulating mtDNA quantification. The experiments were repeated at least three times, and the data are shown as the mean ± standard error (N = eight independent cell isolations per group). * p < 0.05.

To investigate the molecular pathways whereby *DNA-PKcs* ablation prevented endothelial dysfunction and microvascular injury during endotoxemic cardiomyopathy, we employed an unbiased approach. Specifically, we performed a proteomic analysis using label-free technology to compare the protein expression profiles of neonatal CMECs isolated from *DNA-PKcs^f/f^/Tie2^Cre^* mice and control *DNA-PKcs^f/f^* mice after lipopolysaccharide treatment.* DNA-PKcs* deficiency altered the expression of 1085 proteins in lipopolysaccharide-treated CMECs (log_2_fold-change > 1.5). A volcano map of the differentially expressed proteins showed that 490 proteins were upregulated and 595 were downregulated in *DNA-PKcs^f/f^/Tie2^Cre^* CMECs (Figure 5A).

A Gene Ontology Biological Process Description analysis indicated that the altered proteins were involved in mitochondrial membrane organization, mitochondrial translation, regulation of mitochondrial membrane potential, and promotion of mitochondrial organization (Figure 5B-C). Similarly, a Gene Ontology Cellular Component Description analysis illustrated that *DNA-PKcs* deficiency primarily influenced proteins in the mitochondrial respiratory chain, mitochondrial protein complex, mitochondrial inner membrane, mitochondrial matrix and mitochondrial envelope (Figure 5B-C). A Kyoto Encyclopedia of Genes and Genomes (KEGG) analysis of these altered proteins showed that mitochondrial metabolic pathways such as lipoic acid metabolism, the tricarboxylic acid cycle, glutathione metabolism, oxidative phosphorylation, and fatty acid elongation were altered as a result of *DNA-PKcs* deficiency in ECs after lipopolysaccharide treatment (Figure 5D). In Gene Set Enrichment Analysis (GSEA) plots, lipopolysaccharide-treated *DNA-PKcs^f/f^/Tie2^Cre^* CMECs exhibited enhanced oxidative phosphorylation, tricarboxylic acid cycling, mitochondrial fusion and mitochondrial DNA (mtDNA) maintenance processes, but reduced mitochondrial fission, compared with lipopolysaccharide-treated control *DNA-PKcs^f/f^* CMECs (Figure 5E-I). In sum, *DNA-PKcs* deficiency seemed to alter the expression of proteins responsible for mitochondrial structure and function in CMECs treated with lipopolysaccharides.

We then performed verification experiments, which confirmed that lipopolysaccharides dissipated the mitochondrial membrane potential in CMECs, whereas *DNA-PKcs* deletion restored it to near-normal levels (Figure 5J). A mitochondrial morphological analysis using immunofluorescence demonstrated that lipopolysaccharide treatment disrupted the mitochondrial network, resulting in fragmented mitochondria in CMECs (Figure 5K-5L). However, *DNA-PKcs* deletion normalized the mitochondrial morphology of CMECs in the presence of lipopolysaccharides (Figure 5K-L). Moreover, we further measured the levels of the circulating mtDNA in *DNA-PKcs^f/f^/Tie2^Cre^* mice and control *DNA-PKcs^f/f^* mice after lipopolysaccharide treatment. The content of circulating mtDNA was significantly elevated in response to the lipopolysaccharide challenge in control *DNA-PKcs^f/f^* mice, whereas this change was markedly suppressed in *DNA-PKcs^f/f^/Tie2^Cre^* mice (Figure 5M). These results suggested that DNA-PKcs impairs mitochondrial structure and function in cardiac microvascular ECs during endotoxemia.

### *DNA-PKcs* ablation upregulates MOTS-c by protecting mitochondria

**Figure 6:**
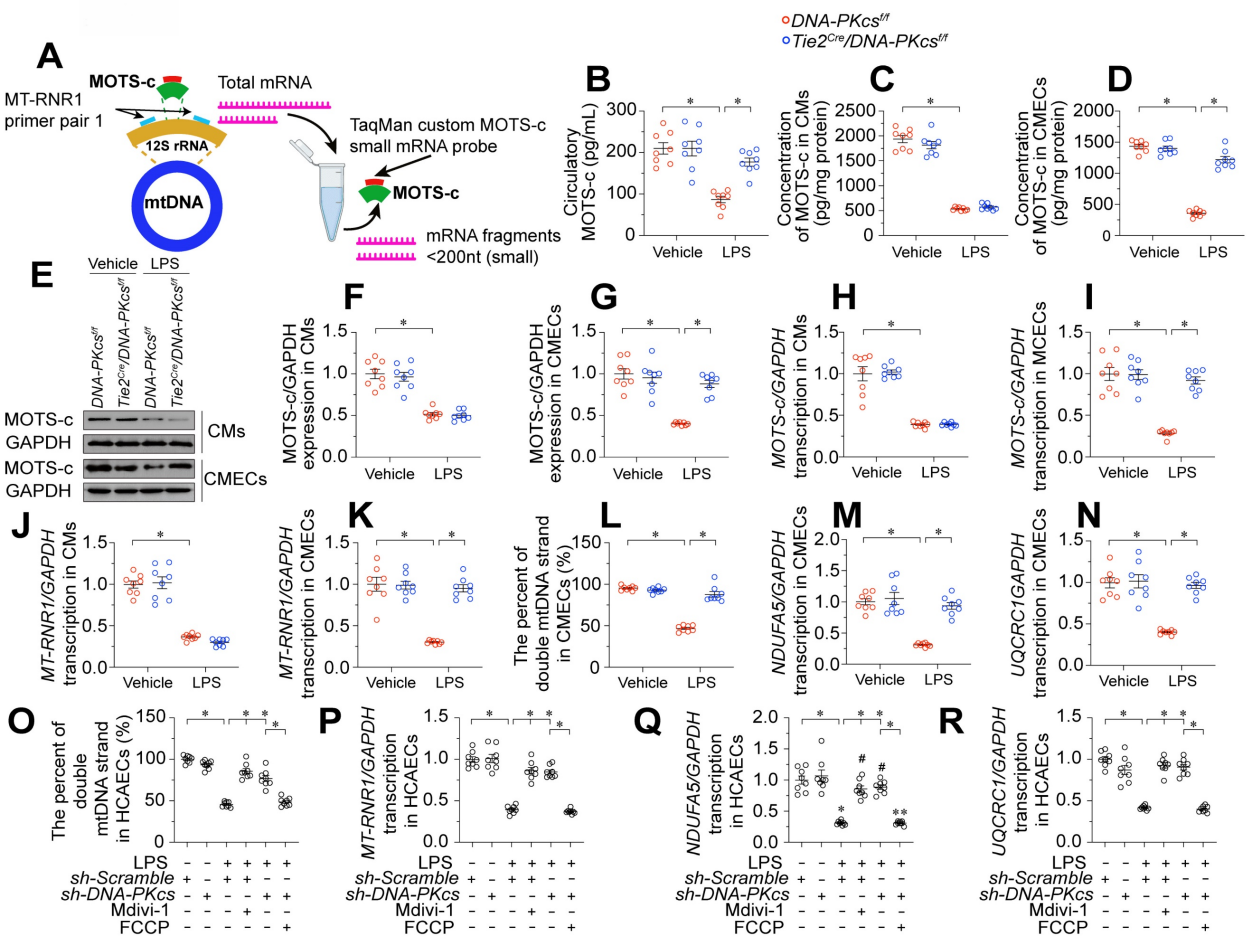
***DNA-PKcs* ablation upregulates MOTS-c by protecting mitochondria.** To induce endotoxemia *in vivo, DNA-PKcs^f/f^* and *DNA-PKcs^f/f^*/*Tie2^Cre^* male mice (eight weeks old) were injected intraperitoneally with a single dose of lipopolysaccharide (10 mg/kg) and evaluated after 72 h. **A.** Analysis technique for 12S rRNA (*MT-RNR1*) mRNA and *MOTS-c* mRNA. **B.** An ELISA kit was used to analyze circulating MOTS-c levels in mice after lipopolysaccharide treatment. **C, D.** CMECs and cardiomyocytes (CMs) were isolated from *DNA-PKcs^f/f^* and *DNA-PKcs^f/f^*/*Tie2^Cre^* male mice and then incubated with lipopolysaccharides. An ELISA was used to determine the concentration of MOTS-c in the medium. **E-G.** Proteins were isolated from CMECs or CMs with or without lipopolysaccharide treatment, and MOTS-c expression was determined using Western blotting. **H, I.** RNA was isolated from CMECs or CMs, and *MOTS-c* mRNA expression was determined using qPCR. **J, K.** qPCR analysis of *MT-RNR1* in CMECs or CMs with or without lipopolysaccharide treatment. **L.** Fluorometric analysis of double-stranded mtDNA breaks, expressed as a percentage of double-stranded mtDNA. **M, N.** The transcript levels of mtDNA were determined based on NADH:ubiquinone oxidoreductase subunit A5 (*NDUPA5*) and ubiquinol-cytochrome c reductase core protein 1 (*UQCRC1*) mRNA expression. **O.** HCAECs were treated with Mdivi-1 (10 nM) or FCCP (30 μM) to inhibit or activate mitochondrial fission, respectively. A fluorometric analysis was performed to assess double-stranded mtDNA breaks in HCAECs, and the results were expressed as a percentage of double-stranded mtDNA **P.** A qPCR analysis was conducted to assess *MT-RNR1* levels in HCAECs **Q-R.** The transcript levels of mtDNA were determined based on *NDUPA5* and *UQCRC1* mRNA expression. Experiments were repeated at least three times and the data are shown as mean ± SEM (N = 8 mice or eight independent cell isolations per group). * p < 0.05.

MOTS-c is a mitochondria-derived peptide encoded in the 12S rRNA region of the mitochondrial genome (Figure 6A). Reduced MOTS-c levels have been regarded as an early sign of mitochondrial dysfunction in a range of cardiovascular diseases, including heart failure 17, diabetic cardiomyopathy 41, and coronary artery disease 42. Thus, we asked whether the mitochondrial protective effects of *DNA-PKcs* ablation were followed by MOTS-c upregulation. We found that lipopolysaccharide-injected control *DNA-PKcs^f/f^* mice had lower circulating MOTS-c levels than vehicle-treated mice (Figure 6B). However, plasma MOTS-c levels were restored to near-physiological concentrations in lipopolysaccharide-treated *DNA-PKcs^f/f^/Tie^Cre^* mice (Figure 6B).

Since MOTS-c is abundant in muscular organs 16, we wondered whether the high plasma levels of MOTS-c in *DNA-PKcs^f/f^/Tie^Cre^* mice originated from ECs or cardiomyocytes. To examine this, we isolated neonatal cardiomyocytes and CMECs from *DNA-PKcs^f/f^* and *DNA-PKcs^f/f^/Tie^Cre^* mice, and treated them with lipopolysaccharides. Lipopolysaccharide treatment rapidly reduced MOTS-c levels in the media from both *DNA-PKcs^f/f^* mouse-derived cardiomyocytes (Figure 6C) and *DNA-PKcs^f/f^* mouse-derived CMECs (Figure 6D). However, while MOTS-c production was normalized in *DNA-PKcs^f/f^/Tie^Cre^* mouse-derived CMECs after the lipopolysaccharide challenge, this normalization did not occur in cardiomyocytes from *DNA-PKcs^f/f^/Tie^Cre^* mice (Figure 6C-D). Intracellular protein analyses using Western blotting similarly showed that MOTS-c expression was restored to near-normal levels after lipopolysaccharide treatment in *DNA-PKcs^f/f^/Tie^Cre^* CMECs, but not in *DNA-PKcs^f/f^/Tie^Cre^* cardiomyocytes (Figure 6E-G), suggesting that EC-specific *DNA-PKcs* deletion stabilized MOTS-c expression in ECs during endotoxemia. Likewise, quantitative polymerase chain reaction (qPCR) analyses showed that *MOTS-c* mRNA levels were substantially reduced by lipopolysaccharide treatment in both *DNA-PKcs^f/f^* cardiomyocytes and *DNA-PKcs^f/f^* CMECs, and were partially restored in *DNA-PKcs^f/f^/Tie^Cre^* CMECs, but not in *DNA-PKcs^f/f^/Tie^Cre^* cardiomyocytes (Figure 6H-I).

Since the coding sequence for *MOTS-c* is found within the 12S rRNA gene (*MT-RNR1*) of mtDNA, reduced *MOTS-c* transcription may result from impaired* MT-RNR1* transcription. To investigate this possibility, we filtered total RNA to enrich small mRNA fragments (< 200 nucleotides), and used a custom TaqMan small RNA assay with a probe designed against the location of the *MT-RNR1* gene (Figure 6A). As expected, *MT-RNR1* transcript levels were substantially lower in lipopolysaccharide-treated *DNA-PKcs^f/f^* cardiomyocytes and *DNA-PKcs^f/f^* CMECs than in the vehicle-treated groups (Figure 6J-K). However, lipopolysaccharide failed to suppress *MT-RNR1* transcription in *DNA-PKcs^f/f^/Tie^Cre^* CMECs, but not in *DNA-PKcs^f/f^/Tie^Cre^* cardiomyocytes (Figure 6J-K).

The 12S rRNA gene (*MT-RNR1*) is encoded within mtDNA; therefore, we asked whether impaired transcription of this gene was secondary to mtDNA damage. Our data indicated that lipopolysaccharide induced double-stranded mtDNA breaks (Figure 6L) and reduced mtDNA transcription (Figure 6M-N) in *DNA-PKcs^f/f^* CMECs. However, *DNA-PKcs* deficiency maintained the integrity of mtDNA and favored mtDNA transcription in CMECs after lipopolysaccharide treatment.

To understand the molecular processes by which lipopolysaccharide induced mtDNA damage and suppressed *MOTS-c* transcription, we focused on pathological mitochondrial fission. Our recent study identified DNA-PKcs as a novel inducer of mitochondrial fission 23, and fragmented mitochondria have been reported to contain broken mtDNA 43. Administration of Mdivi-1, an inhibitor of mitochondrial fission, was able to prevent lipopolysaccharide-induced double-stranded mtDNA breaks (Figure 6O), maintain *MT-RNR1* transcription (Figure 6P) and restore *MOTS-c* mRNA expression (Figure 6Q-R) in *DNA-PKcs^f/f^* CMECs. On the other hand, activation of mitochondrial fission through supplementation with carbonyl cyanide-p-(trifluoromethoxy) phenylhydrazone (FCCP) was associated with mtDNA breaks (Figure 6O), reduced *MT-RNR1* transcription (Figure 6P) and diminished *MOTS-c* mRNA expression in lipopolysaccharide-treated *DNA-PKcs^f/f^/Tie^Cre^* CMECs (Figure 6Q-R). Thus, by activating DNA-PKcs, lipopolysaccharide promoted excessive mitochondrial fission, which disrupted the mitochondrial genome and reduced *MOTS-c* expression.

### MOTS-c downregulation is associated with increased coronary injury and endothelial dysfunction

To assess the clinical relevance of MOTS-c levels in endotoxemia-induced myocardial coronary injury and heart dysfunction, we examined circulating CD34^+^ ECs and EPCs isolated from human septic patients. Western blotting and qPCR analyses showed that MOTS-c protein and mRNA levels in circulating CD34^+^ ECs (Figure S2A-B) and EPCs (Figure S2C-D) were significantly lower in septic patients than in healthy subjects. Moreover, lower MOTS-c levels in EPCs were associated with higher APACHE II scores, SOFA scores and lactic acid levels, but lower LVEF values (Table S7). FMD, endo-PAT, CF-PWV, CAVI and ABI measurements also demonstrated the clinical association of reduced EPC MOTS-c expression with cardiac endothelial dysfunction (Table S7). These results suggested that reduced MOTS-c expression contributes to cardiac coronary injury and endothelial dysfunction during endotoxemia.

### MOTS-c supplementation attenuates endotoxemia-induced myocardial microvascular damage and endothelial barrier dysfunction

**Figure 7:**
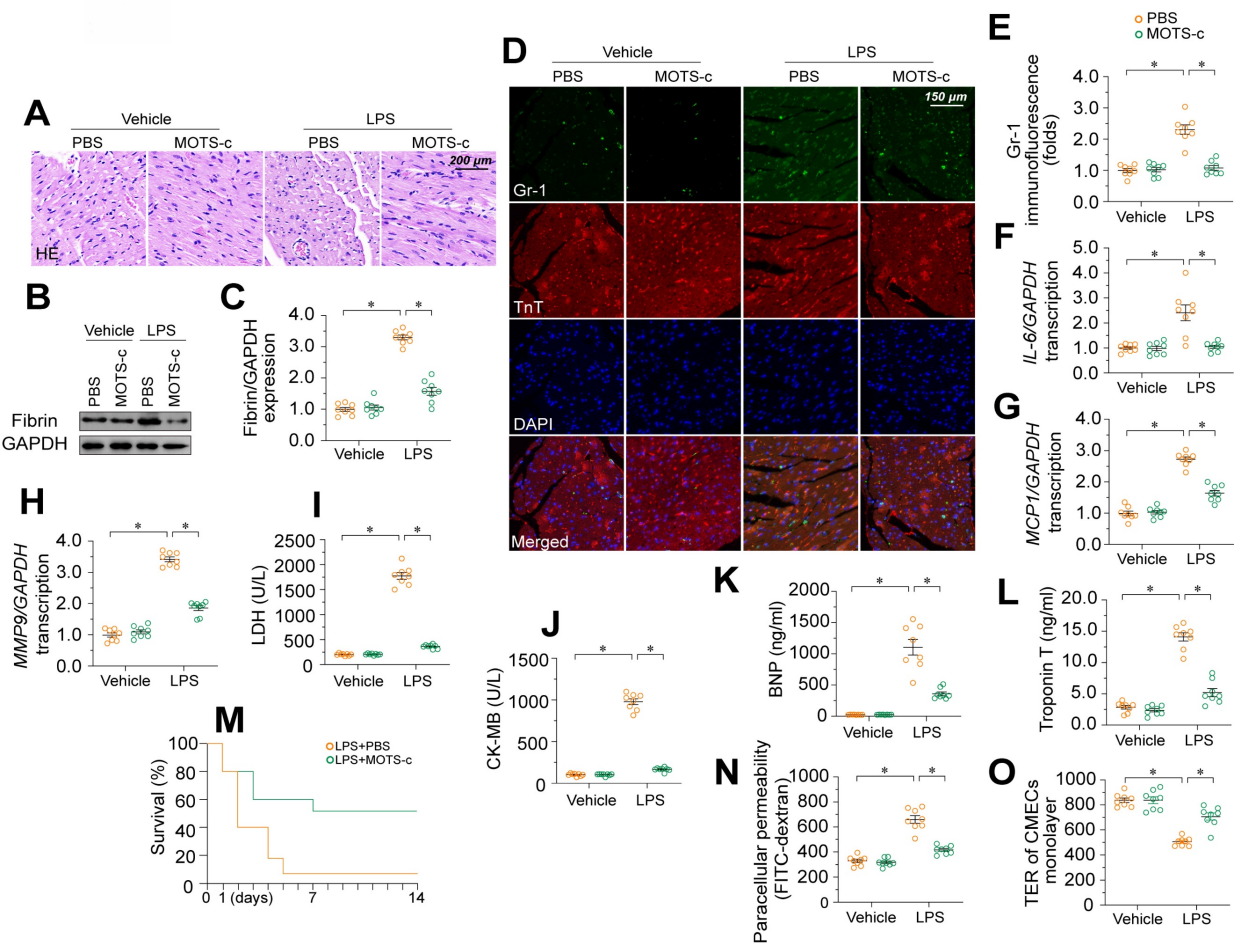
** MOTS-c supplementation attenuates endotoxemia-induced myocardial microvascular damage and endothelial barrier dysfunction.** To induce endotoxemia *in vivo*, WT mice were injected intraperitoneally with a single dose of lipopolysaccharide (10 mg/kg) and evaluated after 72 h. MOTS-c (15 mg/kg) was administered daily via intraperitoneal injection for seven days before lipopolysaccharide-induced endotoxemia. *In vitro*, HCAECs were treated with 10 μg/mL lipopolysaccharide for 24 h. MOTS-c (10 µM) or the vehicle (PBS) was added to the medium 24 h before the application of lipopolysaccharide stress. **A.** H&E staining of erythrocyte aggregation in microvessels after lipopolysaccharide treatment. **B, C.** Proteins were extracted from CMECs isolated from mice with or without lipopolysaccharide treatment, and fibrin expression was determined using Western blotting. **D, E.** Immunofluorescence staining of GR-1-positive neutrophils in the myocardium. **F-H.** RNA was isolated from heart tissue, and qPCR was used to assess the transcription of *IL-6*, *MCP1,* and *MMP9*. **I-L.** ELISA kit analysis of cardiac injury biomarkers, including serum TnT, CK-MB, LDH, and BNP. **M.** Survival times of different mice in the presence or absence of lipopolysaccharides. **N, O.** FITC clearance and TER assays were performed to detect alterations in the endothelial barrier. Experiments were repeated at least three times and the data are shown as mean ± SEM (N = 8 mice or eight independent cell isolations per group). *p < 0.05.

To further explore the involvement of MOTS-c in endotoxemia-induced myocardial microvascular damage, we injected WT mice with exogenous MOTS-c seven days before lipopolysaccharide treatment. Compared with vehicle injection, MOTS-c administration prevented erythrocyte accumulation (Figure 7A) in the presence of lipopolysaccharides. MOTS-c also nullified lipopolysaccharide-induced fibrin deposition (Figure 7B-C) and GR-1-positive neutrophil recruitment to the myocardium (Figure 7D-E). In addition, MOTS-c repressed pro-inflammatory cytokine upregulation in heart tissues following lipopolysaccharide treatment (Figure 7F-H). Due to these endothelial protective actions, MOTS-c treatment also reduced myocardial injury biomarker levels (Figure 7I-L), improved heart function (Table S8), and prolonged the survival time (Figure 7M) of lipopolysaccharide-treated mice. *In vitro*, MOTS-c-treated HCAECs exhibited lower FITC retention and higher TER values than vehicle-treated HCAECs following lipopolysaccharide exposure (Figure 7N-O).

### MOTS-c downregulation is involved in DNA-PKcs-induced profilin phosphorylation and lamellipodial degradation

**Figure 8:**
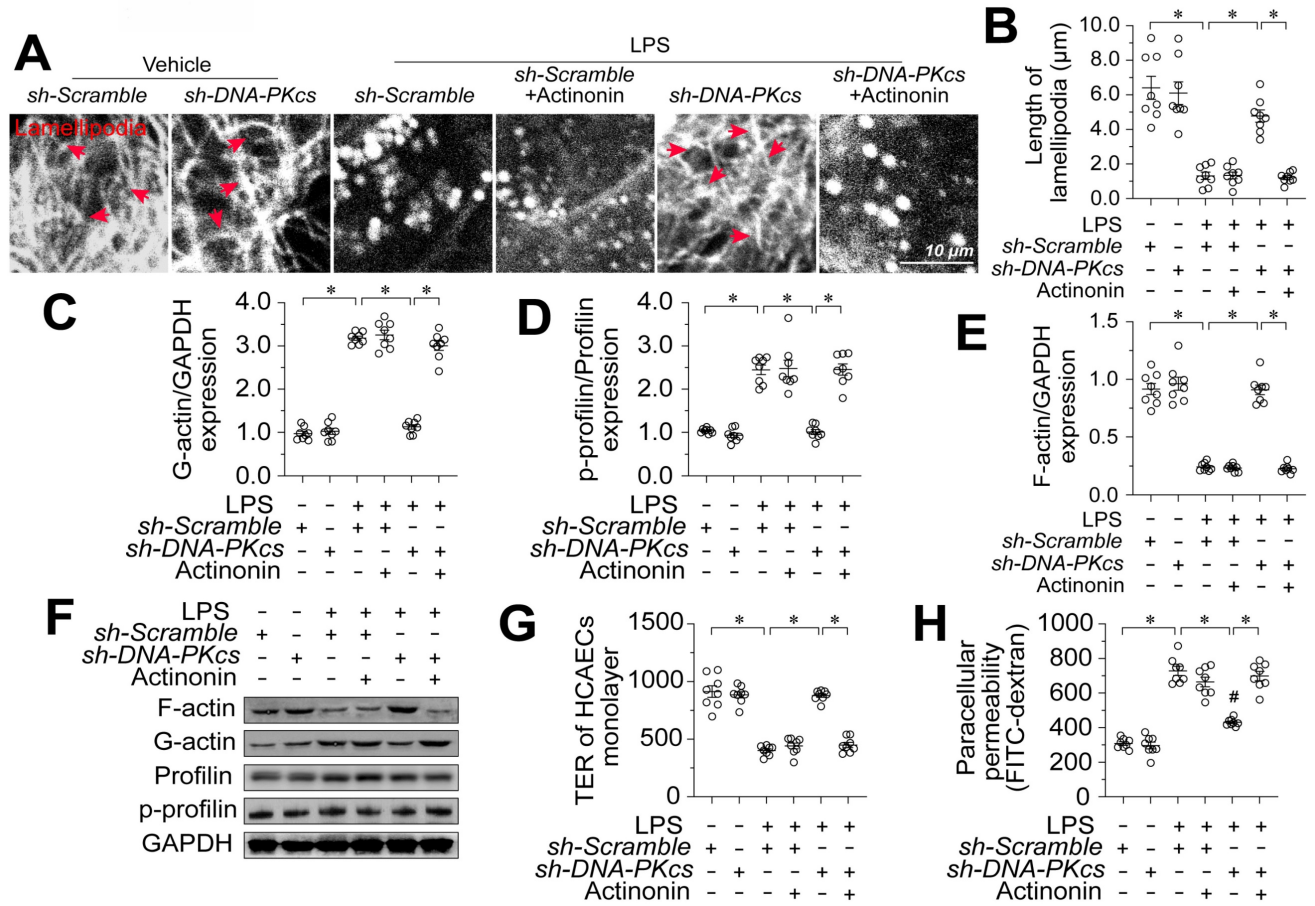
** MOTS-c downregulation is involved in DNA-PKcs-induced profilin phosphorylation and lamellipodial degradation.** *In vitro*, HCAECs were transfected with sh-DNA-PKcs or sh-scramble before being treated with 10 μg/mL lipopolysaccharide. HCAECs were treated with actinonin (20 nM) to deplete mitochondrial RNA. **A, B.** Immunofluorescence assays were used to observe F-actin expression and lamellipodia. The average lamellipodial length was determined. **C-F.** Proteins were isolated from HCAECs, and Western blotting was used to assess G-actin, F-actin, and phosphorylated profilin levels. **G, H.** FITC clearance and TER assays were performed to detect alterations in the endothelial barrier. Experiments were repeated at least three times and the data are shown as mean ± SEM (N = 8 mice or eight independent cell isolations per group). *p < 0.05.

Next, we investigated the involvement of MOTS-c in the protective effects of *DNA-PKcs* deletion on EC barrier integrity. Since targeted knockout or knockdown methods for mitochondrial genes are not available, we selectively depleted mitochondrial RNA in HCAECs using actinonin 37 before transfecting the cells with sh-DNA-PKcs. Then, we measured cytoskeleton, F-actin, and profilin levels in these cells in the presence of lipopolysaccharides. An immunofluorescence assay showed that sh-DNA-PKcs prevented lipopolysaccharide-induced lamellipodial degradation in HCAECs, whereas actinonin nullified this effect (Figure 8A-B). In contrast, neither sh-DNA-PKcs nor actinonin altered tubulin expression (Figure 8A-B). In lipopolysaccharide-treated HCAECs, F-actin expression was reduced, and G-actin levels were increased compared with control cells (Figure 8C-E). Treatment with sh-DNA-PKcs normalized the F-actin/G-actin ratio following lipopolysaccharide exposure but not in HCAECs treated with actinonin (Figure 8C-E). Further, sh-DNA-PKcs attenuated lipopolysaccharide-induced profilin phosphorylation in HCAECs, whereas actinonin counteracted this effect (Figure 8D-F). Lastly, although sh-DNA-PKcs suppressed FITC retention and increased the TER values in lipopolysaccharide-treated HCAECs, these protective effects were absent in HCAECs treated with actinonin (Figure 8G-H). These results suggested that *MOTS-c* upregulation accounted for the beneficial outcomes of *DNA-PKcs* deficiency, such as profilin non-phosphorylation, lamellipodial polymerization, and improved endothelial barrier function.

### MOTS-c prevents profilin phosphorylation by suppressing the JNK pathway

**Figure 9:**
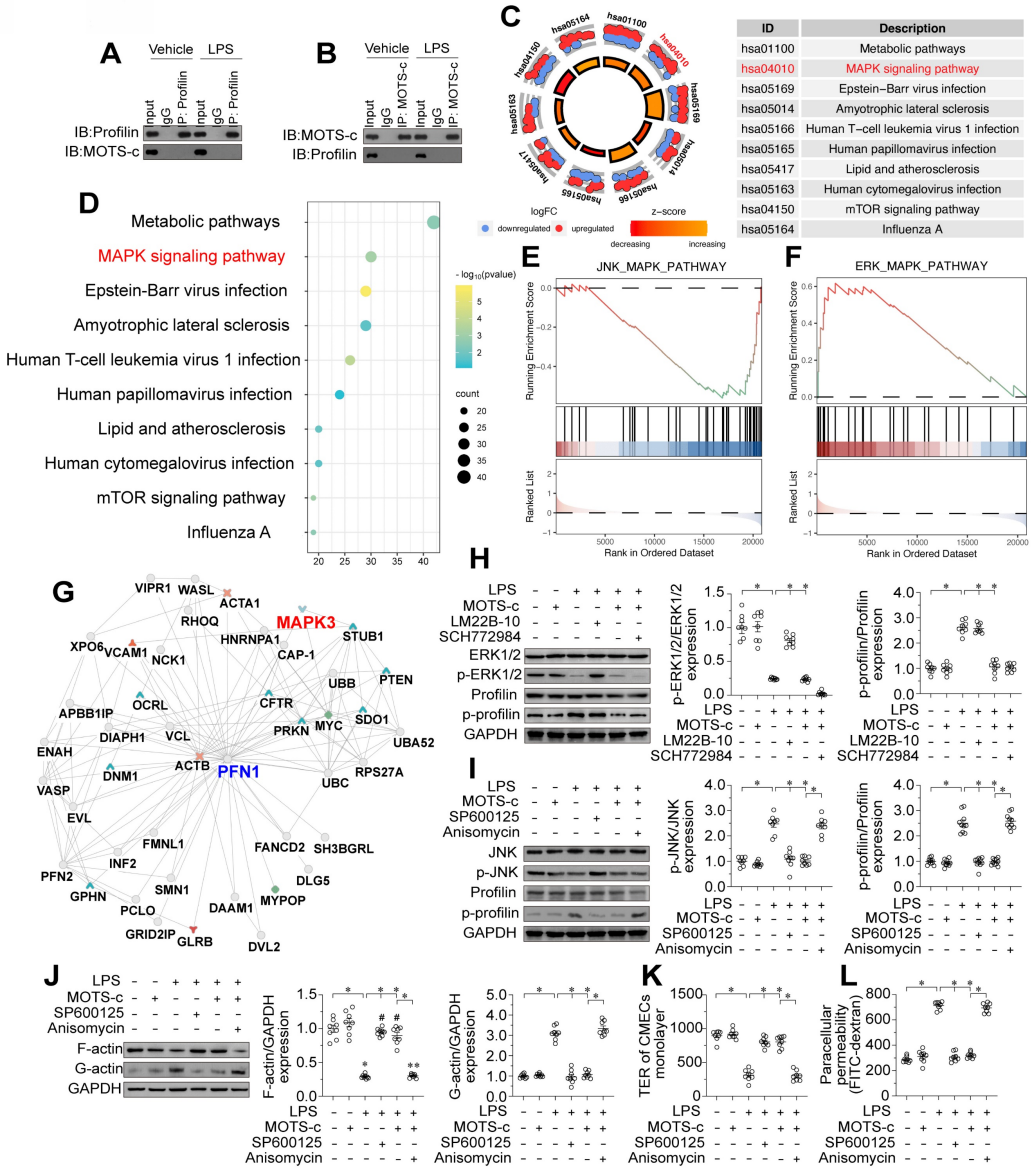
** MOTS-c prevents profilin phosphorylation by suppressing the JNK pathway.** To induce endotoxemia *in vivo,* WT mice were injected intraperitoneally with a single dose of lipopolysaccharide (10 mg/kg), and evaluated after 72 h. *In vitro*, HCAECs were incubated with 10 μg/mL lipopolysaccharide for 24 h. MOTS-c (10 µM) or the vehicle (PBS) was added to the medium 24 h before the application of lipopolysaccharide stress. **A, B.** A co-immunoprecipitation assay was used to detect potential binding between MOTS-c and profilin in HCAECs in the presence of lipopolysaccharides.** C, D.** KEGG analysis of RNA-seq data from HEK293 cells in the presence or absence of MOTS-c. **E, F.** GSEA Visual Analysis of MAPK-related Pathways in HEK293 cells. **G.** The potential protein interactive network of profilin in HEK293 cells was analyzed using the inBio Discover™ database. **H, I.** HCAECs were treated with an ERK1/2 inhibitor (SCH772984, 5 μM), ERK1/2 activator (LM22B-10, 10 μM), JNK inhibitor (SP600125, 10 μM) or JNK activator (anisomycin, 2 μM). Then, proteins were isolated from the cells, and Western blotting was used to evaluate JNK and ERK1/2 expression. **J.** Proteins were isolated from HCAECs, and Western blotting was used to assess F-actin and G-actin expression. **K, L.** FITC clearance and TER assays were performed to detect alterations in the endothelial barrier. Experiments were repeated at least three times and the data are shown as mean ± SEM (N = 8 mice or eight independent cell isolations per group). *p < 0.05.

Our last question was how MOTS-c prevents profilin phosphorylation in ECs. We did not observe direct binding between MOTS-c and profilin in a co-immunoprecipitation assay (Figure 9A-B), suggesting that MOTS-c inhibits profilin phosphorylation through other pathways or kinases. Given that MOTS-c downregulation has been associated with JNK activation 21, we wondered whether profilin phosphorylation in ECs depended on JNK activation resulting from MOTS-c inactivation. To identify potential downstream signaling pathways of MOTS-c, we analyzed RNA-seq data from human embryonic kidney (HEK293) cells with or without exogenous MOTS-c treatment. A KEGG analysis indicated that metabolic pathways and the mitogen-activated protein kinase (MAPK) signaling pathway were potential downstream effectors of MOTS-c (Figure 9C-D). Further analyses demonstrated that the JNK pathway was inhibited after MOTS-c treatment, whereas the extracellular signal-regulated kinase 1/2 (ERK1/2) pathway was activated (Figure 9E-F). We also assessed the potential protein interactive network of profilin using the inBio Discover™ database (https://inbio-discover.com), which suggested that MAPK interacts with profilin (Figure 9G). Considering these findings, together with the fact that MAPK phosphorylates proteins as a kinase, we hypothesized that MOTS-c prevents profilin phosphorylation by altering MAPK family protein expression.

We then performed verification experiments in HCAECs treated with lipopolysaccharides, with or without MOTS-c supplementation. A protein phosphorylation analysis demonstrated that lipopolysaccharide treatment inhibited the ERK1/2 pathway but activated the JNK pathway in HCAECs (Figure 9H-I). MOTS-c administration re-activated the ERK1/2 pathway but suppressed the JNK pathway in lipopolysaccharide-treated HCAECs (Figure 9H-I). In addition, we examined the alterations in signaling pathways of other members within the MAPK family, including p38 MAPK and ERK5. Western blot analysis revealed that the levels of phosphorylated p38 and phosphorylated ERK5 were significantly increased upon lipopolysaccharide stimulation, while treatment with MOTS-c had no effect on their phosphorylation (Figure S3A-D). These findings indicate that p38, ERK1/2, and ERK5 are not downstream signaling targets of MOTS-c, whereas JNK is regulated by MOTS-c in the presence of lipopolysaccharides. This was further corroborated by ELISA analyses, which demonstrated that lipopolysaccharide-induced activation of p38 and ERK5, as well as the inactivation of ERK1/2, were not modulated by MOTS-c treatment (Figure S3E-H). Conversely, the activation of JNK induced by lipopolysaccharides could be effectively mitigated by MOTS-c (Figure S3E-H).

Interestingly, the application of an ERK1/2 inhibitor (SCH772984) prevented MOTS-c from activating ERK1/2 but did not prevent MOTS-c from maintaining profilin dephosphorylation in lipopolysaccharide-treated HCAECs (Figure 9H-I). In contrast, treatment of lipopolysaccharide-supplemented HCAECs with a JNK activator (anisomycin) prevented MOTS-c from both inactivating JNK and maintaining profilin dephosphorylation (Figure 9H-I). Moreover, a JNK inhibitor (SP600125), but not an ERK1/2 activator (LM22B-10), was able to repress profilin phosphorylation in lipopolysaccharide-treated HCAECs (Figure 9H-I). These results demonstrated that MOTS-c inhibited profilin phosphorylation by impeding JNK activation in lipopolysaccharide-treated ECs.

We also evaluated the involvement of JNK signaling in endothelial barrier function. Blocking the JNK pathway promoted lamellipodial formation in HCAECs under lipopolysaccharide stress, possibly by normalizing the F-actin/G-actin ratio (Figure 9J). Moreover, the JNK inhibitor SP600125 ameliorated lipopolysaccharide-induced FITC retention and TER reduction in HCAECs (Figure 9K-L). On the other hand, in lipopolysaccharide-treated HCAECs, the JNK activator anisomycin negated the protective effects of MOTS-c on endothelial barrier functional parameters such as F-actin polymerization (Figure 9J), TER maintenance (Figure 9K) and FITC clearance (Figure 9L). Overall, MOTS-c preserved endothelial barrier function by inactivating the JNK pathway upon lipopolysaccharide stress.

## Discussion

**Figure 10:**
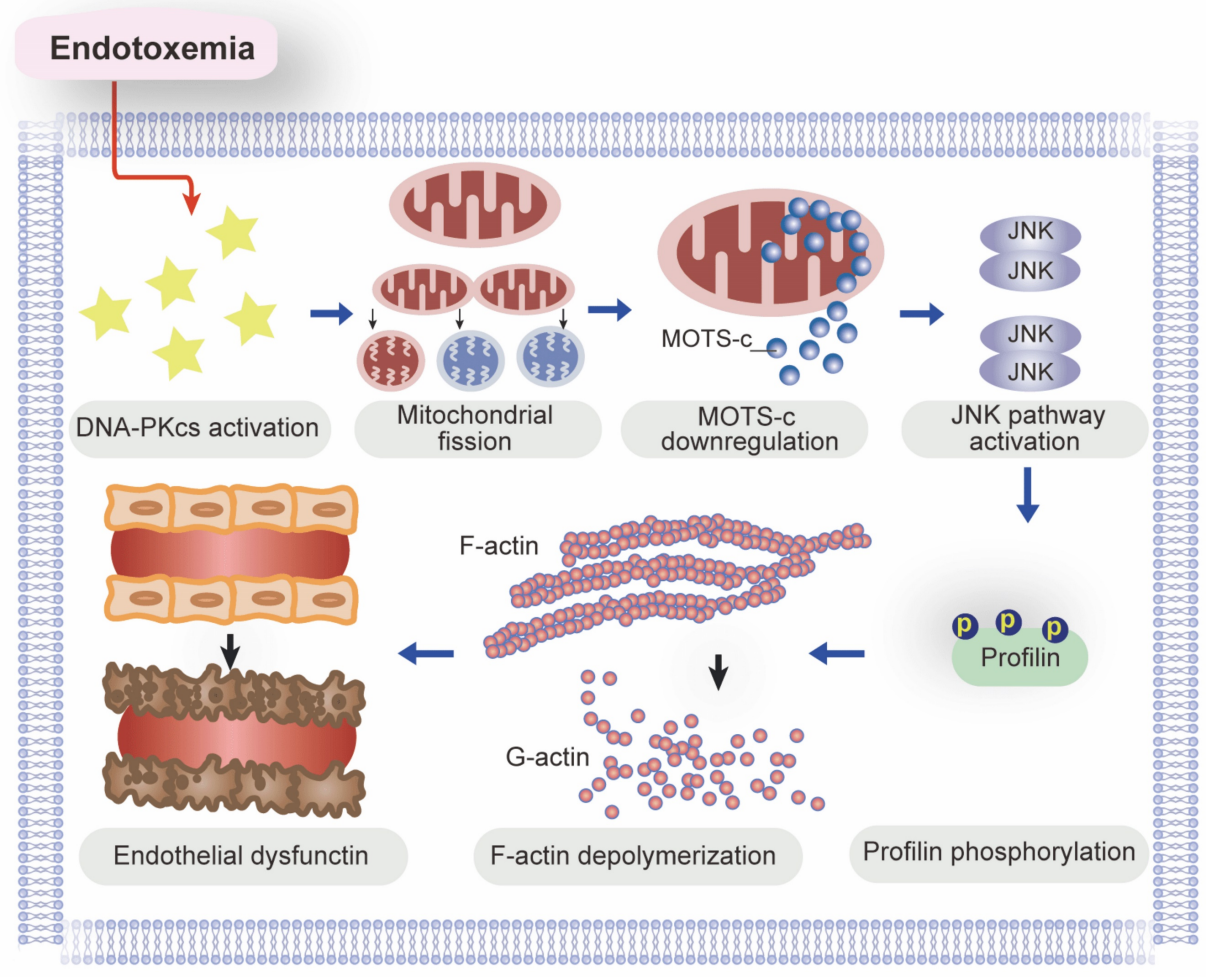
A schematic mechanism of DNA-PKcs-mediated MOTS-c downregulation as well as profilin-mediated lamellipodia degradation in the pathogenesis of endotoxemia-induced myocardial microvascular injury.

In this study, we used genetic modification methods and pharmacological interventions *in vivo* and *in vitro* to explore the molecular pathways behind endotoxemia-induced endothelial dysfunction and myocardial microvascular injury. Our study had three main findings (Figure 10). First, DNA-PKcs activation is a newly discovered pathological contributor to lipopolysaccharide-induced microvascular damage, as it undermines lamellipodia-dependent endothelial barrier integrity. Second, DNA-PKcs activation disrupts mitochondrial homeostasis in ECs by inducing excessive mitochondrial fission, resulting in mtDNA breaks that impair the transcription of *MOTS-c*. Third, due to its downregulation under lipopolysaccharide stress, MOTS-c fails to inhibit JNK activation and thus promotes JNK-dependent profilin phosphorylation in ECs, leading to F-actin depolymerization and lamellipodial degradation. According to our findings in ECs, DNA-PKcs deciphers signals from the lipopolysaccharide stimulus and transmits these detrimental messages to mitochondria, inducing mitochondrial genomic breaks that reduce the expression of *MOTS-c*. When produced at adequate levels, MOTS-c suppresses JNK activity, thus protecting ECs against lipopolysaccharide-induced lamellipodial depolymerization, barrier dysfunction and microvascular disorder. In view of these results, inhibiting DNA-PKcs activation and/or enhancing MOTS-c expression are potential therapeutic approaches to prevent JNK-dependent profilin phosphorylation and protect the endothelial barrier against endotoxemia.

DNA-PKcs is known to be involved in DNA damage repair as part of the non-homologous end-joining pathway. However, the DNA damage response has been identified as a maladaptive response to inflammation, and there is a complex relationship between DNA-PKcs and inflammation. DNA-PKcs can bind to various transcription factors, including nuclear factor-κB 44 and signal transducer and activator of transcription 3 45, which are key regulators of inflammation. Moreover, ablation of *DNA-PKcs* has been reported to attenuate the synthesis and release of IL-6 and IL-12 in dendritic cells 46. DNA-PKcs influences the activities of not only pro-inflammatory pathways and cytokines but also immune cells such as macrophages 47 and T cells 48. In fact, DNA-PKcs has been identified as an intracellular sensor of lipopolysaccharide stress in macrophages 49. Similarly, our recent observations demonstrated that lipopolysaccharides activated DNA-PKcs in the liver, kidneys 23, and heart 22. The current study revealed additional pro-inflammatory properties of DNA-PKcs in myocardial microvessels and suggested that DNA-PKcs inactivation could be a worthwhile therapeutic strategy to interrupt pro-inflammatory signal transduction during endotoxemia-induced microvascular injury.

In this study, we identified aberrant activation of DNA-PKcs as a key upstream signal precipitating mitochondrial dysfunction in ECs during endotoxemia. This study primarily examines a select few critical lipopolysaccharide-induced mechanisms responsible for the decline in mitochondrial function, but other multiple molecular pathways associated with endotoxemia can also contribute to reduced mitochondrial efficacy. Oxidative stress, a known factor in mitochondrial damage, can be elicited by lipopolysaccharides and mitigated by mitochondria-targeted antioxidants to preserve endothelial barrier integrity in lipopolysaccharide-induced acute lung injury 50. Additionally, a reduction in nitric oxide (NO) synthesis has recently been proposed as a mechanism fostering mitochondrial fragmentation in ECs. This occurs via the modulation of manganese superoxide dismutase (MnSOD) stability 51. Furthermore, anomalous calcium signaling, attributed to diminished sarco(endo)plasmic reticulum calcium-ATPase 2 (SERCA2) activity during endotoxemia, is linked with Drp1-mediated mitochondrial fission 52. Compounding these factors, endotoxemia-induced hypoxia and inflammation typically co-occur, disrupting gas exchange and consequentially leading to hypoxia. This hypoxic state suppresses Toll-like receptor 4 (TLR4), culminating in impaired mitochondrial electron transport and compromised mitochondrial respiration 53. Intriguingly, the mitochondrial redistribution of uncoupled endothelial nitric oxide synthase (eNOS), contingent upon RohA-Rho-associated coiled-coil containing protein kinase (ROCK) signaling, is intimately associated with inflammasome activation and apoptosis in ECs during acute lung injury 54. Combining these insights with our findings suggests that protecting mitochondria could be a viable approach to mitigate lipopolysaccharide-mediated dysfunction in ECs.

MOTS-c was recently discovered as a mitochondria-derived peptide with various beneficial effects, especially anti-inflammatory properties. MOTS-c has been found to inhibit the production of pro-inflammatory cytokines such as tumor necrosis factor α and IL-6 55, and to suppress the nuclear factor-kB pathway 56. The present study revealed that MOTS-c downregulation correlated with JNK pathway activation in ECs, in accordance with previous studies showing that MOTS-c inhibited JNK function in formalin-induced inflammatory nociception 19, lipopolysaccharide-induced acute lung injury 57 and lipopolysaccharide-induced septic cardiomyopathy 58. Interestingly, one study found that MOTS-c did not suppress JNK in neuroinflammation 59, suggesting that the post-transcriptional modification of JNK by MOTS-c varies among cell types or disease models.

The prominent finding of our study was the molecular pathway through which lipopolysaccharides suppressed MOTS-c expression in ECs. *MOTS-c* is encoded by mtDNA, so its transcriptional downregulation can result from mitochondrial genomic injury 21. Mitochondria are dynamic organelles, and mitochondrial morphological alterations influence their genomic stability. Previous studies 60, 61 and our recent findings 62, 63 have highlighted the cause-effect relationship between mitochondrial fission and mtDNA damage; thus, *MOTS-c* downregulation may occur secondary to mitochondrial fission. DNA-PKcs is a kinase, Fis1 is a confirmed substrate of DNA-PKcs 23, and the relationship between DNA-PKcs activation and mitochondrial fission has been well-established. Based on the above evidence, we speculated that DNA-PKcs downregulates *MOTS-c* by inducing mitochondrial fission in ECs, and our results confirmed this hypothesis. Therefore, inhibiting mitochondrial fission and protecting the stability of the mitochondrial genome are vital for maintaining *MOTS-c* abundance in ECs. Lastly, since exogenous administration of MOTS-c was sufficient to attenuate endotoxemia-induced myocardial microvascular injury, MOTS-c supplementation could be an endothelium-specific therapeutic approach to improve myocardial microvascular function during endotoxemia.

Profilin is a small actin-binding protein that is involved in various cellular processes, including cytoskeletal organization and cell motility 64. Several studies have illustrated the necessity of profilin for maintaining endothelial barrier integrity 65-67. The function of this barrier largely depends on cell-cell junctions formed by EC lamellipodia 68, which are highly dynamic actin-based structures. Profilin determines the extent of lamellipodial formation by facilitating F-actin polymerization 69, 70. Previous studies have demonstrated the adverse effects of profilin phosphorylation on F-actin integrity and lamellipodial formation 71-73; however, the upstream kinase responsible for profilin phosphorylation had not been elucidated. Our research addressed this issue and revealed that the JNK pathway, which is negatively regulated by MOTS-c, is necessary for profilin phosphorylation in ECs. It remains to be determined whether the JNK/profilin pathway influences other endothelial functions, such as adhesion, anticoagulation, and angiogenesis.

In this study, we described a novel pathological signaling pathway responsible for endotoxemia-induced endothelial barrier dysfunction and myocardial microvascular injury. We found that lipopolysaccharide exposure activated DNA-PKcs and thus promoted mitochondrial fission in ECs, leading to mitochondrial genomic damage and reduced transcription of mtDNA-encoded *MOTS-c*. Due to its downregulation in ECs, MOTS-c failed to prevent lipopolysaccharide-induced JNK activation, resulting in profilin phosphorylation, F-actin depolymerization, lamellipodial degradation, and endothelial barrier dysfunction. Based on these findings, we propose DNA-PKcs inhibition and MOTS-c supplementation as promising therapeutic options to protect the endothelium and myocardial microvessels against endotoxemia.

## Supplementary Material

Supplementary figures and tables.
