# Supplementary material for: The DNA-dependent protein kinase catalytic subunit exacerbates endotoxemia-induced myocardial microvascular injury by disrupting the MOTS-c/JNK pathway and inducing profilin-mediated lamellipodia degradation
Source: Theranostics. 2024 Feb 4;14(4):1561–82. doi: 10.7150/thno.92650 (PMC10879869; doi:10.7150/thno.92650)
Supplement: Supplementary file 1 — Supplementary figures and tables. [file thnov14p1561s1.pdf]

Supplemental Figures and Tables

The DNA-dependent protein kinase catalytic subunit exacerbates endotoxemia-induced myocardial microvascular injury by disrupting the MOTS-c/JNK pathway and inducing profilin-mediated lamellipodia degradation

Supplemental Table 1. Demographics of septic patients diagnosed with (+) or without (-) septic cardiomyopathy (SC)

Patient characteristics	SC (-) patients (n=174)	SC (+) patients (n=66)
Age	64.9±15.8	77.7±15.2
Sex	Male (n=89)	Male (n=30)
Body weight (kg)	61.9±11.4	62.4±9.4
Comorbidities (n)		
Atrial fibrillation	8	25
Heart failure	13	28
Diabetes	9	34
Hypertension	4	12
Tumor	3	7
Coronary artery disease	35	7
Echocardiography		
Ejection fraction (%)	69.2±4.7	43.1±6.4
Mitral annular plane systolic excursion (mm)	15.1±2.7	9.8±3.7
Tricuspid annular plane systolic excursion (mm)	21.3±2.7	13.8±4.1
Fractional area change (%)	50.2±8.6	36.1±11.4
Pathogen		
Pseudomonas aeruginosa	51 (29.31%)	18 (27.27%)
Klebsiella pneumoniae	46 (26.43%)	15 (22.73%)
Enterobacter aerogenes	31 (17.82%)	10 (15.15%)
Proteus mirabilis	23 (13.22%)	8 (12.12%)
Escherichia coli	20 (11.50%)	7 (10.61%)
Acinetobacter baumannii	16 (9.20%)	6 (9.10%)
Stenotrophomonas maltophila	11 (6.32%)	4 (6.10%)
Enterococcus faecalis	10 (5.75%)	4 (6.10%)
Staphylococcus aureus	4 (2.30%)	2 (3.03%)

Staphylococcus epidermidis	2 (1.15%)	1 (1.52%)
Candida albicans	3 (1.72%)	2 (3.03%)
Clinical data		
Maximum body temperature (°C)	37.7±1.8	38.3±1.2
White blood cells (x10^9/L)	10.5±1.9	12.8±2.7
C-reactive protein (mg/dL)	6.5±0.9	22.7±5.5
Procalcitonin (ng/dL)	7.2±1.9	12.6±2.7
Platelets (x10^9/L)	152.1±99.4	87.5±69.4
Lactic acid (mmol/L)	1.9±0.24	4.86±1.92
APACHE II score	19±4	27±8.2
SOFA score	7.1±2.3	13.4±3.2

Supplemental Table 2. Antibody information

Name	Catalogue number	Dilution factor
DNA-PKcs	Abcam, #ab32566	1:1000
p-DNA-PKcs	Abcam, #ab18192	1:1000
Profilin	Abcam, #ab124904	1:1000
p-Profilin	Abcam, #ab215752	1:1000
F-actin	Abcam, #ab205	1:1000
Albumin	Abcam, #ab192603	1:1000
JNK	Abcam, #ab307802	1:1000
p-JNK	Abcam, #ab307802	1:1000
ERK	Abcam, #ab184699	1:1000
p-ERK	Abcam, #ab192591	1:1000
GR-1	Abcam, #ab25377	1:1000
TnT	Abcam, #ab8295	1:1000
G-actin	Abcam, #ab123034	1:1000
eNOS	Abcam, #ab300071	1:1000
p-eNOS	Abcam, #ab215717	1:1000
MOTS-c	MyBioSource, #MBS542112	1:1000
Fibrin	Creative-biolabs, #MOB-0417ZL	1:1000

Supplemental Table 3. Primers for qPCR

Gene	Forward Primer	Reverse Primer
Mouse *Il-6*	5'-CAGACTCGCGCCTCTAAGGAGT-3'	5'-GATAGCCGATCCGTCGAA-3'
Mouse *Mcp1*	5'-GATAGCCGATCCGTCGAA-3'	5′-GCTACCACAACATCTGGACATT-3′
Mouse *Mmp9*	5′-AACCAATGATGCTGGGTTCAC-3′	5'-GCGCCGACTCAGAGGTGT-3'
Mouse *18S*	5′-TAGAGGGACAAGTGGCGTTC-3′	5'-CGCTGAGCCAGTCAGTGT-3'
Mouse *mt-COI*	5′-GCCCCAGATATAGCATTCCC-3′	5′-GTTCATCCTGTTCCT GCTCC-3′
Human *NDUFA5*	5′-ATCACCTTCGAGAAGCTGGA-3′	5'-ACTTCACCACCCTGAAGCAA-3'
Human *UQCRC1*	5′-GCCGGGGCACAAGTGCTAT-3′	5'-CTTGGACAGCGCCTTGATGT-3'
Human *MT-RNR1*	5′-AGCGCAAGTACCCACGTAAA-3′	5'-AGGGCCCTGTTCAACTAAGC-3'
Mouse *Gapdh*	5′-TCGATATTGAGCGTCCAACCT-3′	5′-CAAAGGCACGTTTGGCATACA-3′
Human *GAPDH*	5′-CCACTCCTCCACCTTTGACG-3′	5′- CCACCACCCTGTTGCTGTAG -3′

Supplemental Table 4. DNA-PKcs activity in EPCs from septic intensive care unit patients. DNA-PKcs activity was analyzed with an ELISA kit. The association between DNA-PKcs activity and heart dysfunction was measured.

Parameters	Low DNA-PKcs activity (n=59)	High DNA-PKcs activity (n=181)	p-value
Heart rate (bpm)	89±12	104±17	<0.001
Average arterial pressure (mmHg)	84±12	84±13	0.463
Central venous pressure (mmHg)	8.4±1.2	9.3±1.4	0.631
Ejection fraction (%)	66.5±4.3	45.2±7.6	<0.001
Mitral annular plane systolic excursion (mm)	14.5±2.9	10.9±3.3	<0.001
Tricuspid annular plane systolic excursion (mm)	20.4±3.1	15.2±3.6	<0.001
Fractional area change (%)	47.7±10.8	39.2±11.9	<0.001
Troponin I (μg/L)	0.03±0.01	0.27±0.09	<0.001
NT-ProBNP (ng/L)	998.5±376.8	4413.6±1755.2	<0.001
Arterial blood lactate (mmol/L)	1.7±0.6	2.5±0.8	0.004
APACHE II score	20±3	24±3	0.015
SOFA score	10.2±2.5	12.4±2.6	0.026
FMD	7.7±0.9	6.3±1.2	0.037
endo-PAT RHI	2.02±0.16	1.29±0.23	0.013
CF-PWV	8.44±1.97	9.93±2.13	0.022
CAVI (m/s)	8.24±1.25	11.47±1.02	<0.001
ABI	1.08±0.09	0.87±0.15	<0.001

NT-ProBNP, N-terminal pro-B-type natriuretic peptide.

Supplemental Table 5. Echocardiographic determination of heart function in *DNA-PKcs*$^{f/f}$ and *DNA-PKcs*$^{f/f}$/*Tie2*^{Cre} mice in the presence of lipopolysaccharide.

Parameters	Vehicle		Lipopolysaccharide	
	DNA-PKcs$^{f/f}$	*DNA-PKcs*$^{f/f}$/*Tie2*^{Cre}	*DNA-PKcs*$^{f/f}$	*DNA-PKcs*$^{f/f}$/*Tie2*^{Cre}
FS, %	33.7±1.5	33.9±1.9	20.9±1.4*	29.9±2.1$^{\#}$
EF, %	62.4±4.7	63.5±4.2	40.8±3.7*	56.8±3.3$^{\#}$
IVS, mm	0.81±0.03	0.80±0.04	0.69±0.02*	0.79±0.04$^{\#}$
PW, mm	0.77±0.06	0.76±0.04	0.76±0.03	0.78±0.04
E/A	1.29±0.26	1.34±0.17	0.88±0.09*	1.15±0.16$^{\#}$

EF, ejection fraction; IVS, interventricular septal thickness; PW, posterior wall thickness; FS, ratio of left ventricular fractional shortening; E/A, ratio of early to late ventricular filling velocities. *$p<0.05$ vs. vehicle+*DNA-PKcs*$^{f/f}$, $^{\#}p<0.05$ vs. lipopolysaccharide+*DNA-PKcs*$^{f/f}$.

Supplemental Table 6. Echocardiographic determination of heart function in WT mice treated with NU7441 in the presence of lipopolysaccharide.

Parameters	Vehicle		Lipopolysaccharide	
	PBS	NU7441	PBS	NU7441
FS, %	34.1±1.8	34.0±1.7	20.8±1.2*	31.6±1.7#
EF, %	63.2±4.1	64.1±3.9	41.2±3.5*	60.2±3.7#
IVS, mm	0.83±0.04	0.82±0.03	0.68±0.04*	0.80±0.02#
PW, mm	0.78±0.04	0.79±0.05	0.77±0.05	0.79±0.05
E/A	1.35±0.22	1.31±0.24	0.91±0.14*	1.25±0.19#

EF, ejection fraction; IVS, interventricular septal thickness; PW, posterior wall thickness; FS, ratio of left ventricular fractional shortening; E/A, ratio of early to late ventricular filling velocities. *$p<0.05$ vs. vehicle+PBS, #$p<0.05$ vs. lipopolysaccharide+PBS.

Supplemental Table 7. The association between MOTS-c expression and heart dysfunction in EPCs isolated from septic intensive care unit patients. MOTS-c was analyzed using Western blotting.

Parameters	Low MOTS-c expression (n=62)	High MOTS-c expression (n=179)	p-value
Heart rate (bpm)	87±11	103±19	<0.001
Average arterial pressure (mmHg)	85±12	84±12	0.586
Central venous pressure (mmHg)	8.6±1.1	9.5±1.3	0.572
Ejection fraction (%)	68.2±5.7	44.3±8.2	<0.001
Mitral annular plane systolic excursion (mm)	14.7±3.1	10.4±2.7	<0.001
Tricuspid annular plane systolic excursion (mm)	20.9±3.6	14.5±2.7	<0.001
Fractional area change (%)	48.3±9.5	36.5±12.5	<0.001
Troponin I (μg/L)	0.03±0.01	0.25±0.11	<0.001
NT-ProBNP (ng/L)	924.8±314.2	4742.1±1925.3	<0.001
Arterial blood lactate (mmol/L)	1.6±0.4	2.8±0.7	<0.001
APACHE II score	19±3	25±3	<0.001
SOFA score	9.8±1.7	13.2±2.4	0.011
FMD	8.1±0.7	6.1±1.1	<0.001
endo-PAT RHI	2.21±0.09	1.17±0.15	<0.001
CF-PWV	7.92±1.56	10.35±1.96	0.0013
CAVI (m/s)	7.95±1.33	12.10±1.15	<0.001
ABI	1.09±0.21	0.85±0.13	<0.001

NT-ProBNP, N-terminal pro-B-type natriuretic peptide.

Supplemental Table 8. Echocardiographic determination of heart function in WT mice treated with MOTS-c in the presence of lipopolysaccharide.

Parameters	Vehicle		Lipopolysaccharide	
	PBS	MOTS-c	PBS	MOTS-c
FS, %	33.9±1.6	33.6±1.8	21.3±1.4*	30.7±1.6#
EF, %	64.3±3.8	63.1±3.2	42.4±3.1*	58.6±2.9#
IVS, mm	0.84±0.05	0.83±0.06	0.65±0.05*	0.79±0.04#
PW, mm	0.79±0.05	0.78±0.05	0.76±0.04	0.77±0.05
E/A	1.31±0.24	1.28±0.32	0.87±0.12*	1.16±0.24#

EF, ejection fraction; IVS, interventricular septal thickness; PW, posterior wall thickness; FS, ratio of left ventricular fractional shortening; E/A, ratio of early to late ventricular filling velocities. *$p<0.05$ vs. vehicle+PBS, #$p<0.05$ vs. lipopolysaccharide+PBS.

Supplemental Figures

Supplemental Figure 1

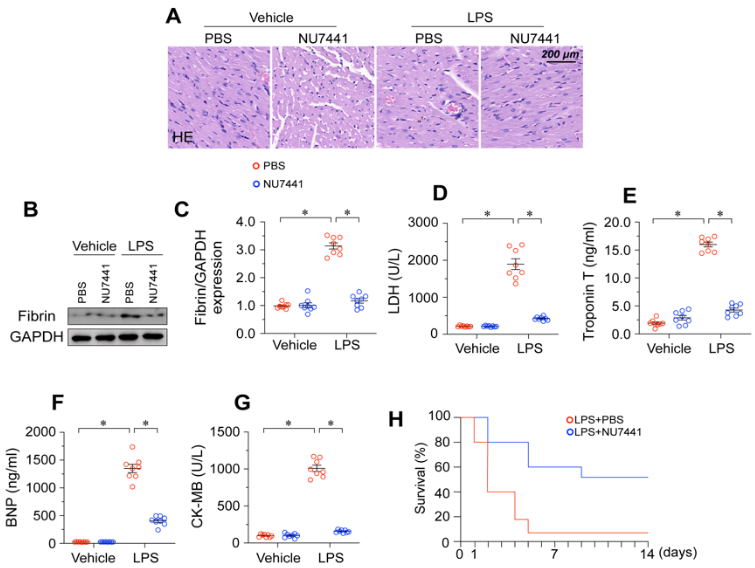

Supplemental Figure 1

DNA-PKcs inhibition attenuates endotoxemia-induced myocardial coronary endothelial injury and heart dysfunction. WT mice were injected intraperitoneally with a single dose of lipopolysaccharide (10 mg/kg) to induce endotoxemia *in vivo*, and were evaluated after 72 hrs. The mice were injected with NU7441 (1 mg/kg) three days before lipopolysaccharide-induced endotoxemia. **A.** H&E staining of erythrocyte aggregation in microvessels after lipopolysaccharide treatment. **B, C.** Proteins were extracted from MCECs isolated from mice with or without lipopolysaccharide treatment, and fibrin expression was determined using Western blotting. **D-G.** ELISA kit analysis of cardiac injury biomarkers (serum TnT, CK-MB, LDH and BNP). **H.** Survival times of different mice in the presence or absence of lipopolysaccharide. *$p<0.05$.

Supplemental Figure 2

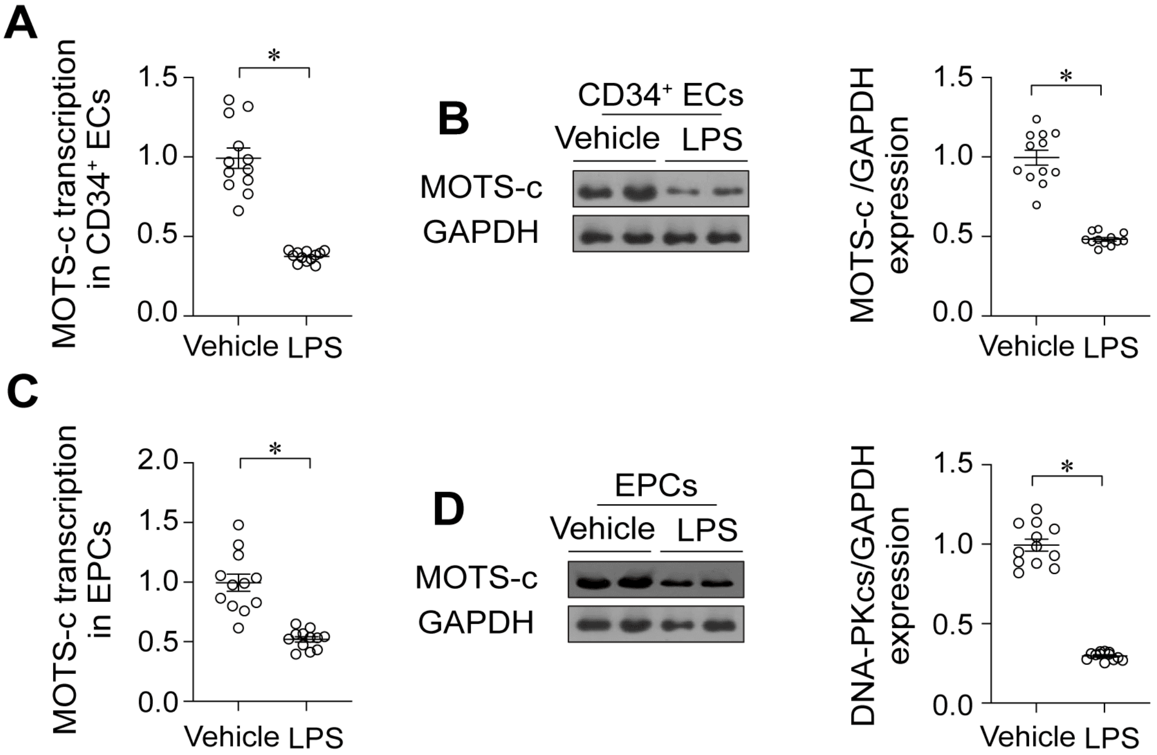

Supplemental Figure 2

MOTS-c downregulation is associated with increased coronary injury and endothelial dysfunction. A-D. $CD34^+$ ECs and EPCs were isolated from septic patients' blood samples using flow cytometry. Then, qPCR was used to analyze *MOTS-c* transcription in $CD34^+$ ECs or EPCs. Western blotting analysis of MOTS-c expression in $CD34^+$ ECs or EPCs isolated from septic patients. *p<0.05.

Supplemental Figure 3

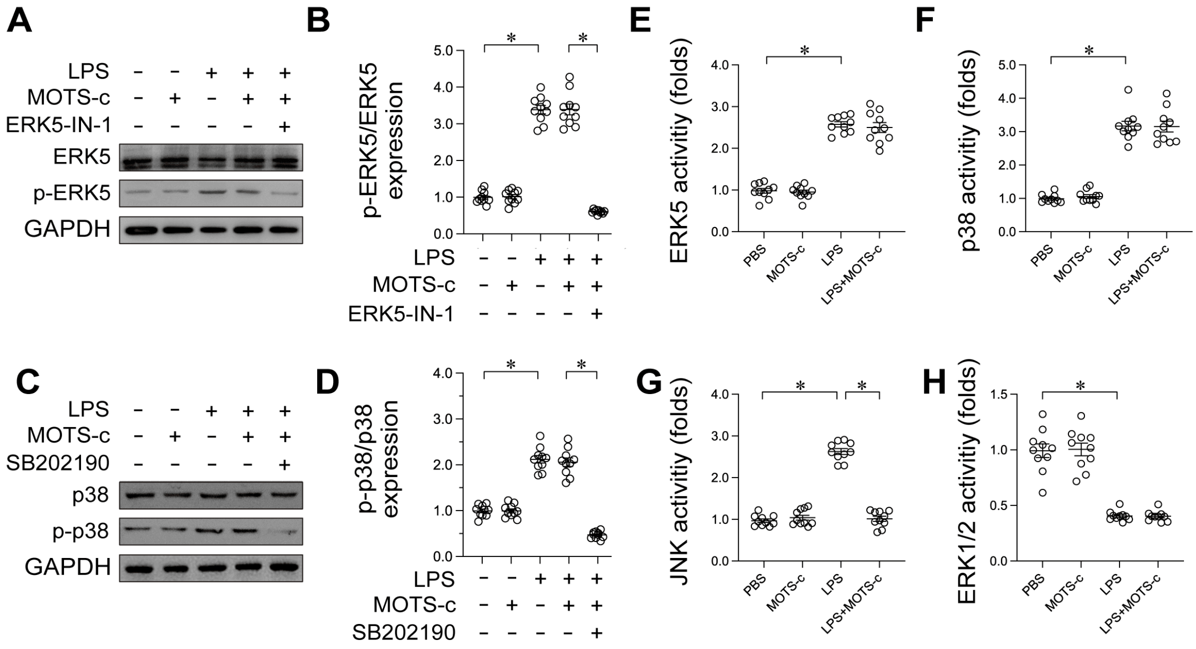

Supplemental Figure 3

MOTS-c have no effects on ERK5 and p38. A-D. *In vitro*, HCAECs were incubated with 10 μg/mL lipopolysaccharide for 24 hrs. MOTS-c (10 μM) or the vehicle (PBS) was added to the medium 24 hrs before lipopolysaccharide stress. ERK5-IN-1 (10 μM, Selleck, cat. no.: S7334) and SB202190 (5 μM, Selleck, cat. no.: S1077) were used to incubate with HCAECs to inhibit ERK5 and p38, respectively. Then, proteins were isolated from the cells, and Western blotting was used to evaluate p38 and ERK5 expression. **E-H.** ELISA kits were used to analyze the activities of ERK5, p38, JNK and ERK1/2 in HCAECs. *$p<0.05$.
